# ACTN1 promotes HNSCC tumorigenesis and cisplatin resistance by enhancing MYH9-dependent degradation of GSK-3β and integrin β1-mediated phosphorylation of FAK

**DOI:** 10.1186/s13046-023-02904-w

**Published:** 2023-12-07

**Authors:** Li Cui, Ye Lu, Jiarong Zheng, Bing Guo, Xinyuan Zhao

**Affiliations:** 1 Stomatological Hospital, School of Stomatology, Southern Medical University, Guangzhou, 510280 Guangdong China; 2 Department of Dentistry, the First Affiliated Hospital, Sun Yat-Sen University, Guangzhou, 510080 China

**Keywords:** ACTN1, HNSCC, Drug resistance, MYH9, Tumorigenesis

## Abstract

**Background:**

Head and neck squamous cell carcinoma (HNSCC) is one of the most common malignant tumors globally. Understanding the molecular basis of tumor progression and drug resistance can offer innovative strategies to enhance clinical outcomes for HNSCC patients.

**Methods:**

The cytoskeletal remodeling genes associated with cisplatin resistance were screened using a PCR array. The role of alpha-actinin 1 (ACTN1) in modulating cisplatin resistance and tumorigenesis in HNSCC was evaluated both in vitro and in vivo*.* Co-immunoprecipitation (Co-IP), IP-mass spectrometry (MS), western blotting, dual-luciferase assay, and bioinformatics analysis were performed to elucidate the underlying mechanisms involved.

**Results:**

Our study identifies ACTN1 as a crucial contributor to cisplatin resistance and tumorigenesis in HNSCC, as evidenced across cellular, animal, and patient-derived xenograft models. From a clinical perspective, overexpression of ACTN1 significantly correlates with a suboptimal response to neoadjuvant chemotherapy and reduced overall survival in HNSCC patients. Mechanistically, ACTN1 predominantly activates β-catenin-mediated signaling by promoting the interaction between myosin heavy chain 9 (MYH9) and GSK-3β, leading to the ubiquitin-dependent degradation of GSK-3β. ACTN1 also interacts with integrin β1, subsequently activating the FAK/PI3K/AKT pathway, providing an additional avenue for the activation of β-catenin signaling. Our study also unveils that the β-catenin/c-Myc axis transcriptionally regulates ACTN1, thereby creating a positive feedback loop promoting HNSCC tumorigenesis and drug resistance.

**Conclusions:**

These insights underscore the novel mechanisms that highlight ACTN1's pivotal role in driving HNSCC progression and resistance to chemotherapy, suggesting ACTN1 as a promising therapeutic target in HNSCC management.

**Supplementary Information:**

The online version contains supplementary material available at 10.1186/s13046-023-02904-w.

## Background

Head and neck squamous cell carcinoma (HNSCC) is ranked as the sixth most common cancer worldwide, contributing significantly to global cancer-related mortality [1, 2]. Despite considerable advancements in multimodal treatment, the overall survival rate for HNSCC remains at 50% [3, 4]. Major barriers to successful HNSCC treatment include innate or acquired resistance to therapies, recurrence of the disease, and metastasis. Consequently, there is an urgent need for a more profound understanding of the molecular mechanisms behind HNSCC, and the development of innovative therapeutic targets to increase the effectiveness of current treatments.

Cytoskeletal remodeling is increasingly recognized as a pivotal factor in cancer progression, particularly regarding therapeutic resistance [5, 6]. Alpha actinins (ACTNs) represent a principal category of actin cross-linking proteins, with four known mammalian isoforms: ACTN1, ACTN2, ACTN3, and ACTN4 [7]. While ACTN2 and ACTN3 are predominantly expressed in muscle tissues, ACTN1 and ACTN4 are ubiquitously present [8]. Notably, ACTN1 mediates a spectrum of critical biological functions, including cell–matrix adhesion, migration, calcium homeostasis, and focal adhesion [9–11]. Dysregulated ACTN1 function has been implicated in the pathogenesis of a diverse range of diseases, such as cancer, rheumatoid arthritis, congenital macrothrombocytopenia, and nemaline myopathy [12–15].

The Wnt/β-catenin signaling pathway, crucial for various physiological processes such as cell proliferation, differentiation, embryonic development, and tissue homeostasis, is closely associated with the onset and progression of diverse cancers when dysregulated [16, 17]. Particularly concerning is the aberrant activation of the Wnt/β-catenin pathway in the context of drug resistance, which has been implicated in the maintenance of cancer stem cells (CSCs) [16]. These CSCs, known as primary drivers of chemoresistance, have been observed to exhibit elevated β-catenin transcriptional activity [18, 19]. The association of β-catenin with chemoresistance underscores the potential of strategies aimed at either targeting β-catenin directly or its upregulators, thereby aiding in the elimination of resistant CSCs, overcoming drug resistance, and enhancing cancer treatment efficacy.

In our study, we identified that ACTN1 is upregulated in cisplatin-resistant HNSCC cell lines and tissues, and its overexpression is associated with poor treatment outcomes. Our findings also reveal that targeting ACTN1 enhances cisplatin sensitivity in HNSCC cells, highlighting its potential as a therapeutic target. Mechanistically, ACTN1 activates β-catenin pathways by promoting GSK-3β degradation through MYH9 interaction and by triggering the FAK/PI3K/AKT pathway through integrin β1 binding. Furthermore, the β-catenin-c-Myc axis regulates ACTN1 expression, establishing a feedback loop that contributes to HNSCC progression and cisplatin resistance.

## Methods

### Patients and clinical samples

This study, which encompassed the use of human tissues and related clinical data, was subject to review and obtained approval from the Institutional Review Board at the First Affiliated Hospital of Sun Yat-sen University (2022–056). Informed consent was provided by all subjects involved. All procedures adhered to the ethical guidelines established in the Declaration of Helsinki. For the cohort of HNSCC patients undergoing induction chemotherapy, the regimen included docetaxel (75 mg/m^2^ on day 1), cisplatin (75 mg/m^2^ on day 1), and 5-fluorouracil (750 mg/m^2^ by infusion over 120 h from day 1 to 5) [20–22]. This standard TPF regimen (docetaxel, cisplatin, and 5-fluorouracil) regimen was administered every three weeks for two cycles. The follow-ups were conducted at regular intervals: during the first two years, patients were monitored by physical examinations every three months. This was followed by biannual examinations during the third to fifth years. After the fifth year, examinations were conducted annually until either the patient’s death or the censoring of their data. The tumor's response to neoadjuvant chemotherapy was assessed using both clinical evaluation and imaging, based on the Response Evaluation Criteria in Solid Tumors (RECIST) guidelines [23, 24]. Clinicopathological parameters, encompassing age, sex, tumor location, smoking history and differentiation status, were extracted from electronic health records. Detailed characteristics of the HNSCC cohort are presented in Supplementary Table 1.

### Cell culture

The HNSCC cell lines UM-SCC-1 (SCC-1) and UT-SCC-23 (SCC-23) were procured from the University of Michigan, while the CAL-27 cell line was purchased from ATCC. The cisplatin-resistant cell lines (SCC-1^cisR^, SCC-23^cisR^, and CAL-27^cisR^) were established following previously described methods [25]. All cell lines were cultivated in Dulbecco's modified Eagle's medium (DMEM), supplemented with 10% fetal bovine serum, 100 U/mL penicillin, and 100 μg/mL streptomycin. The cultures were maintained at 37 °C in a humidified atmosphere containing 5% CO_2_.

### Plasmids, siRNAs, cell transfection, and lentivirus production

SiRNAs targeting c-Myc, MYH9, β-catenin or integrin β1 were synthesized by RiboBio (Guangzhou, China). Transient siRNA transfections were performed using Lipofectamine RNAiMAX (Thermo Fisher Scientific, Waltham, MA USA), while plasmid DNAs were transfected with Lipofectamine 3000 (Thermo Fisher Scientific), as per the manufacturer's instructions. Short hairpin RNA (shRNA) oligonucleotides targeting ACTN1 were inserted into the LV3-pGLV-h1-GFP-puro vector. Concurrently, the full-length human ACTN1 gene was integrated into the pGCL-GFP lentiviral expression vector. Lentiviral particles were produced by transfecting HEK293T cells with the recombinant lentiviral expression vectors and the necessary packaging plasmids. After incubation for 72 h, the lentivirus-containing supernatants were collected and concentrated. The HNSCC cells were transfected with the purified lentiviruses at a multiplicity of infection of 30. Lentiviral particles carrying MYH9 or β-catenin shRNA were sourced from GeneChem (Shanghai, China). The sequences of oligonucleotides used in this study are listed in Supplementary Table S2.

### MTT assay

Cells with the indicated modifications were seeded at a density of 3000 cells per well in 96-well cell culture plates. After an incubation period ranging from one to four days, each well was supplemented with 20 μL of a 5 mg/mL MTT solution at the designated time points. The cells were subsequently incubated for 4 h at 37 °C in a humidified incubator. The supernatant was removed and the formazan product solubilized with 200 μL of dimethyl sulfoxide. Absorbance at 570 nm was measured using a Synergy HT multi-detection reader (Bio-Tek Instruments, Winooski, VT, USA).

### Colony formation assay

Cells subjected to specified treatments were seeded in six-well plates. Following a two-week incubation, the cells were fixed with 4% paraformaldehyde and stained with 0.5% crystal violet.

### Sphere formation assay

Cells were seeded in ultra-low-attachment plates and cultured in DMEM/F12 (Gibco, Grand Island, NY, USA) supplemented with 1% B27 (Invitrogen, Carlsbad, CA, USA), 1% N2 (Invitrogen), 20 ng/mL EGF, and 10 ng/mL bFGF. The culture medium was replaced every two days until tumor spheres developed.

### Matrigel invasion assay

This assay was performed using Matrigel-coated chambers (BD Biosciences, Bedford, MA, USA). Cancer cells with a density of 5.0 × 10^5^ cells per 300 µL of DMEM were placed into the upper chamber, while DMEM supplemented with 10% FBS was added to the lower compartment. After a 24 h incubation, non-invading cells on the upper surface of the Transwell membrane were gently swabbed off. Invading cells were fixed with 4% paraformaldehyde and stained with 0.5% crystal violet. The average invasion area, indicative of cell invasion, was calculated using Image software (Bethesda, Maryland, USA).

### Immunohistochemistry (IHC)

Formalin-fixed and paraffin-embedded tissues were deparaffinized in xylene, rehydrated through graded ethanol, and then washed in phosphate-buffered saline. The tissue sections were treated with 0.3% H_2_O_2_ in methanol to quench endogenous peroxidase activity. Following a 2 h blocking step with goat serum at room temperature, the slides were incubated overnight with primary antibodies at 4 °C, and then with horseradish peroxidase-conjugated secondary antibodies for 1 h at room temperature. Staining signals were visualized using a DAB kit, and slides were counterstained with hematoxylin. After washing with distilled water, the slides were dehydrated with xylene and mounted permanently. Each slide was assessed for staining intensity and assigned an H-score using a specific formula. The H-score is the sum of the products of staining intensity categories and their respective percentages of area stained. Intensity categories are scored as follows: 0 for no staining, 1 for weak staining, 2 for moderate staining, and 3 for strong staining. The H-score is calculated by multiplying the percentage of the area with strong staining by 3, the percentage with moderate staining by 2, and the percentage with weak staining by 1. Areas without staining are multiplied by 0 and thus do not contribute to the score. The final H-score is a cumulative total of these calculations, providing a range from 0 to 300 [26, 27]. Two independent pathologists blinded to the clinical data performed the assessments.

### Western blotting

Tissues and cells were lysed in RIPA buffer (Beyotime, Shanghai, China) containing a protease inhibitor cocktail. Equal amounts of protein were resolved on 4%-20% SDS–polyacrylamide gels, electrophoresed at a constant voltage (150 V) until the tracking dye reached the bottom of the gels. Proteins were transferred to polyvinylidene fluoride membranes using a Trans-Blot Turbo system (Bio-Rad, Hercules, CA, USA). Membranes were blocked in Protein-Free Rapid Blocking Buffer (EpiZyme, Shanghai, China) for 10 min at room temperature, followed by an overnight incubation with primary antibodies at 4 °C. After five washes in TBST, membranes were incubated with HRP-conjugated secondary antibodies for 1 h at room temperature. Following three additional washes with TBST, antibody-antigen complexes were detected using the Amersham ECL Plus Western Blotting Detection Reagent (GE Healthcare, Chicago, IL, USA). Primary antibodies specific for the following proteins were used in this study: ACTN1 (Proteintech, Chicago, IL, USA), GAPDH (Proteintech), SNAI1 (Cell Signaling Technology, Danvers, MA, USA), SNAI2 (Proteintech), TWIST1 (Proteintech), ZEB1 (Proteintech), Vimentin (Proteintech), E-cadherin (Proteintech), N-cadherin (Proteintech), Cyclin D1 (Proteintech), β-catenin (Proteintech), c-Myc (Proteintech), MMP-7 (Santa Cruz Biotechnology, Inc., Santa Cruz, CA, USA), PTCH1 (Proteintech), PTCH2 (Abcam, Cambridge, UK), GLI1 (Proteintech), SHH (Cell Signaling Technology), HEY1 (Proteintech), HES1 (Cell Signaling Technology), p-Smad2 (Cell Signaling Technology), Smad2 (Proteintech), p-Smad3 (Cell Signaling Technology), Smad3 (Proteintech), p-FAK (Thermo Fisher Scientific), FAK (Proteintech), p-PI3K (Cell Signaling Technology), PI3K (Cell Signaling Technology), p-AKT (Proteintech), AKT (Proteintech), Integrin β1 (Proteintech), CD44 (Proteintech), MYH9 (Proteintech), GSK-3β (Proteintech), BIRC5 (Proteintech), and epitope tag antibodies (Proteintech).

### Real-time PCR and cytoskeletal remodeling array

Total RNA from cell samples was extracted using the Quick-RNA™ kit (Zymo Research Corp, Irvine, CA, USA), followed by its reverse transcription into cDNA via SuperScript III Reverse Transcriptase (Invitrogen). Real-time PCR was subsequently carried out on a Bio-Rad CFX96 system using Light Cycler 480@ SYBR Green I MasterMix (Roche, Applied Science, Indianapolis, IN, USA). Relative gene expression alterations were calculated using the 2^−ΔΔCT^ method, employing GAPDH as the internal control. The primers used in this study are listed in Supplementary Table 2. For qPCR array analysis, cDNA from the experimental samples was loaded onto a GeneQuery™ Human Cytoskeletal Remodeling qPCR Array (ScienCell Research Laboratories, Carlsbad, CA, USA), and subsequent procedures were performed in accordance with the manufacturer's instructions.

### Apoptosis assay

The cells with indicated treatments were collected and subjected to Annexin V/propidium iodide (PI) staining (Invitrogen) according to the manufacturer’s instructions. Briefly, cells were washed with cold PBS and resuspended in binding buffer. After being stained with Annexin V-APC and PI, cells were then analyzed by flow cytometry.

### Flow cytometry

Stained cells were subjected to analysis utilizing a DxFLEX flow cytometer (Beckman Coulter, Brea, CA, USA). The APC fluorescence signal was detected in the FL6 channel, whereas the PI signal was captured in the FL2 channel. Analytical gating strategies were employed to exclude cellular debris and doublet events. A forward scatter versus side scatter plot was generated to identify the cellular population. Subsequently, an Annexin V-APC versus PI plot was created to categorize cells into viable (Annexin V-negative, PI-negative), early apoptotic (Annexin V-positive, PI-negative), late apoptotic (Annexin V-positive, PI-positive), and necrotic (Annexin V-negative, PI-positive) statuses. The proportion of cells undergoing apoptosis was quantitatively assessed based on these criteria.

### Luciferase reporter assay

To assess the activity of β-catenin signaling, TOPFlash or FOPFlash luciferase reporter vectors were co-transfected with the relevant plasmids into the cancer cells. The TOPFlash and FOPFlash luciferase reporter systems are commonly used for evaluating the canonical Wnt/β-catenin signaling pathway's activity [28]. The TOPFlash reporter plasmid comprises two repeats of three optimal copies of wild-type TCF binding sites, positioned upstream of the thymidine kinase minimal promoter and the luciferase open reading frame. Conversely, the FOPFlash reporter plasmid harbors the same thymidine kinase promoter but with mutated TCF binding sites, along with the same luciferase open reading frame as the TOPFlash reporter plasmid, serving as a negative control for TOPFlash activity. In the context of the c-Myc related reporter assay, the TOPFlash or FOPFlash plasmid was replaced by either the wild-type (pGL3-ACTN1-wt) or the mutant ACTN1 (pGL3-ACTN1-mut) luciferase reporter plasmid. Cell transfection was conducted using Lipofectamine 3000 transfection reagent (Invitrogen) as per the manufacturer's guidelines. Relative luciferase activity was measured 24 h post-transfection utilizing the Dual-Luciferase Reporter Assay System (Promega, Madison, WI, USA).

### Chromatin immunoprecipitation-quantitative polymerase chain reaction (ChIP-qPCR)

The ChIP assay was conducted using the EZ ChIP Chromatin Immunoprecipitation Kit (Millipore, Billerica, MA, USA), adhering to previously outlined methods [25]. Briefly, DNA–protein cross-links were established by treating cell samples with 1% formaldehyde for 10 min at room temperature. Subsequently, samples were washed thrice with PBS, followed by lysis in RIPA buffer. Sonication was then employed to fragment the genomic DNA. The resulting chromatin fragments were immunoprecipitated with an anti-c-Myc antibody at 4 °C overnight. Associated DNA sequences were identified through qPCR. Primer sequences used for ChIP-qPCR are available upon request.

### Clinical relevance of *ACTN1* in HNSCC determined by public databases

Datasets GSE127165, GSE143224, GSE26549, GSE6631, GSE37991, GSE58911, GSE25099, GSE34105, GSE55550, GSE23558, GSE30784, GSE65858, GSE41613, GSE30788, GSE117973, GSE27020, GSE23036, GSE40774, GSE136037 and GSE85195 were retrieved from the Gene Expression Omnibus (GEO) repository of the NCBI (https://www.ncbi.nlm.nih.gov/geo/). For the TCGA HNSCC cohort, RNA-seq data along with corresponding clinical details were procured from the National Cancer Institute Genomic Data Commons portal (https://gdc.cancer.gov/). Survival analysis was performed by employing X-tile software (https://medicine.yale.edu/lab/rimm/-research/software/) to define the optimal cutoff for separating HNSCC patients into high and low *ACTN1* expression cohorts. Gene Set Enrichment Analysis (GSEA) was performed after segregating patients into *ACTN1*-high and *ACTN1*-low groups based on the median value of *ACTN1* expression.

### Co-immunoprecipitation (Co-IP) assay

Cells subjected to indicated modifications were lysed with RIPA buffer. Post-centrifugation at 14,000 × g for 20 min at 4 °C, the supernatant was incubated overnight with primary antibodies at 4 °C. The resulting immunocomplexes were then coupled to prewashed magnetic beads (EpiZyme) for 6 h at 4 °C to form antigen–antibody-bead complexes. Following three washes with elution buffer, the complexes were denatured at 100 °C for 10 min and subsequently analyzed via western blotting.

### Liquid chromatography with tandem mass spectrometry (LC–MS/MS)

Proteins interacting with ACTN1 were separated via SDS-PAGE. Subsequently, candidate bands were digested with sequencing-grade trypsin (Promega) and submitted to MS/MS analysis for protein identification.

### Cycloheximide (CHX) chase assay

The indicated cells were treated with 100 μg/mL CHX to block de novo protein synthesis. At designated time points post-treatment, total protein lysates were collected and subjected to western blotting to assess GSK-3β degradation rate.

### Animal experiments

All animal procedures complied with the guidelines of the Institutional Animal Care and Use Committee (IACUC) at Southern Medical University. Six-week-old BALB/c nude male mice served as the in vivo model for the subcutaneous tumor study. A volume of 100 μL containing a single-cell suspension at a density of 2 × 10^7^ cells/mL was subcutaneously injected into the dorsal skin of each mouse. The mice were subsequently euthanized four weeks post-injection, and the weight and volume of the resulting tumors were recorded. Following this, tumor tissues were fixed, embedded in paraffin, and prepared for subsequent IHC analysis. To establish patient-derived xenograft (PDX) mouse models, surgical specimens from the primary tumors of HNSCC patients were dissected into small fragments (2–3 mm^3^) and subcutaneously transplanted into NSG mice within 4 h post-resection. Routine monitoring of body weight, tumor growth, and overall health status was performed. Upon reaching a volume of approximately 1 cm^3^, the tumors were excised, and the animals were euthanized. These tumors were serially transplanted into fresh NSG mice, initiating the creation of passage 1 (P1) PDX tumors. This procedure was repeated for the generation of passage 2 (P2) PDX tumors. We successfully established three distinct PDX models (PDX-1, PDX-2, and PDX-3), each derived from a unique HNSCC patient, for subsequent analyses. To evaluate the effects of both ACTN1 depletion and cisplatin on PDX growth, primary tumor cells from PDX-1 were transduced with the corresponding lentiviral vectors (shCTRL, shACTN1 #1). An equal number of these transduced cells were subsequently implanted subcutaneously into NSG mice. The mice were then categorized into four groups (*n* = 6): shCTRL, shACTN1 #1, cisplatin (5 mg/kg, administered intraperitoneally), and shACTN1 #1 plus cisplatin. The treatment period lasted for four weeks. To assess the potential of shACTN1, alone or in combination with cisplatin, to influence the tumorigenicity of HNSCC cells, primary tumor cells were isolated from PDX1 tissues collected from mice in different treatment groups. These cells were then implanted into mice at varying cell counts (1 × 10^4^, 1 × 10^5^). The number of mice that developed tumors was evaluated, and the CSC frequency was determined using the Extreme Limiting Dilution Analysis software (https://bioinf.wehi.edu.au/software/elda/).

### Statistical analysis

Statistical analyses were performed with GraphPad Prism 9.0 (GraphPad Software, San Diego, CA, USA). One-way analysis of variance and Student's t-tests were applied to analyze group differences. Data are reported as means ± standard deviations unless otherwise indicated. Kaplan–Meier method was utilized to estimate survival probabilities, and survival distributions were compared using the log-rank test. The degree of association between two variables was quantified using the Pearson correlation coefficient. *P* values less than 0.05 were considered statistically significant.

## Results

### ACTN1 is a key driver of cisplatin resistance in HNSCC

**Fig. 1:**
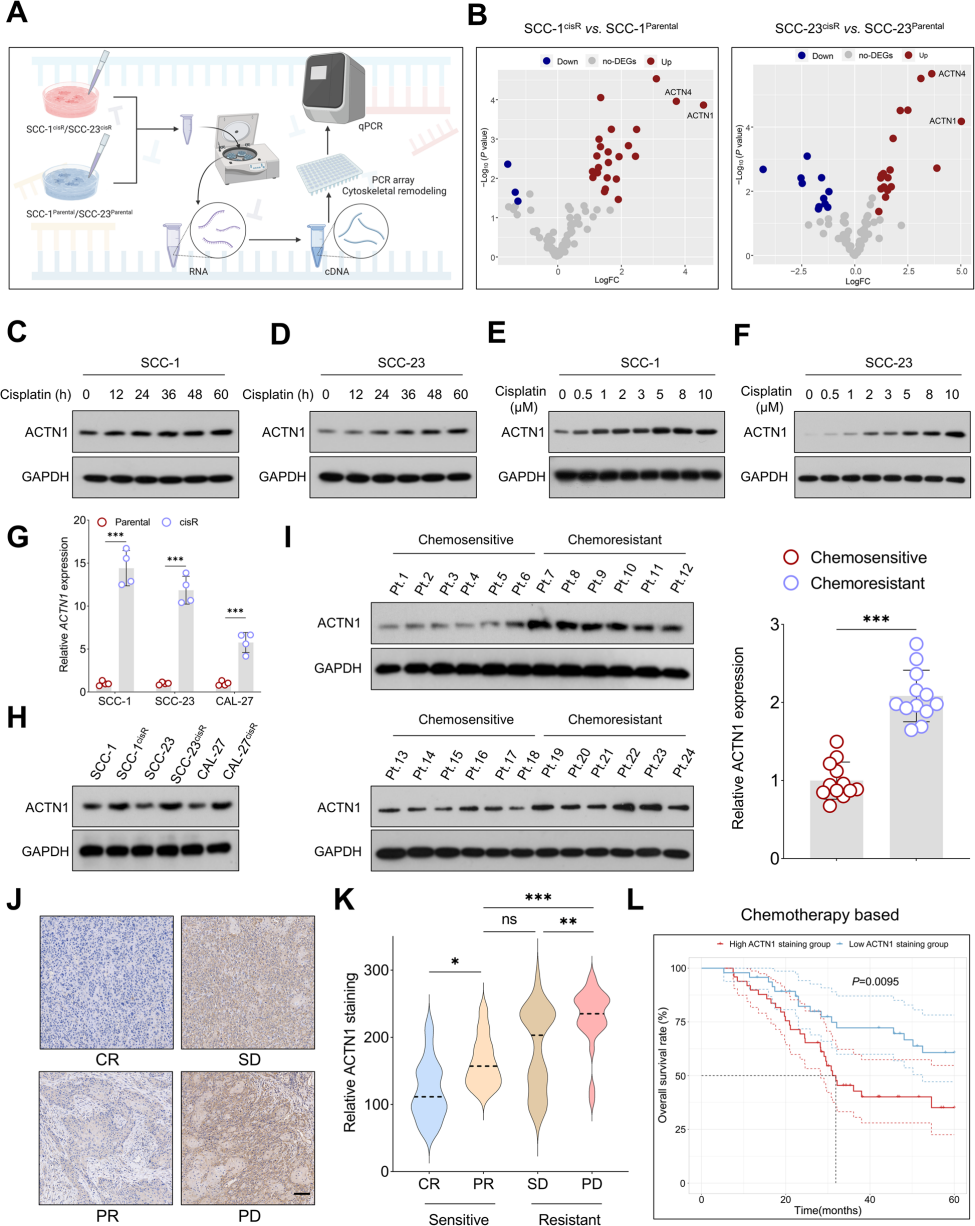
ACTN1 is a critical factor in cisplatin resistance in HNSCC. **A** Schematic diagram illustrating the PCR array experimental workflow. **B** Volcano plots representing differentially expressed genes between SCC-1^cisR^/SCC-23.^cisR^ cells and their respective parental controls. **C-D** Western blot analysis of ACTN1 expression in HNSCC cells exposed to cisplatin over varying durations. **E–F** Western blot analysis of ACTN1 expression in HNSCC cells subjected to varying doses of cisplatin for 48 h. **G-H** qPCR and western blot analyses of ACTN1 expression in cisplatin-resistant HNSCC cells compared to parental control cells. **I** Western blot analysis of ACTN1 expression in both chemosensitive and chemoresistant HNSCC tissues. **J-K** Correlation between the staining intensity of ACTN1 in an in-house HNSCC cohort before neoadjuvant chemotherapy and the therapeutic efficacy. **L** Survival analysis of chemotherapy-treated patients at baseline, stratified by the staining intensity of ACTN1. *P<0.05, **P<0.01, ***P<0.001, ns: not significant

To identify key cytoskeletal remodeling genes associated with cisplatin resistance, we initially compared gene expression differences between cisplatin-resistant cells and their parental counterparts using a PCR array. This analysis revealed that *ACTN1* and *ACTN4* were the most significantly enriched genes in both SCC-1^cisR^ and SCC-23^cisR^ cells (Fig. 1A-B), suggesting a potential correlation with cisplatin resistance in HNSCC cells. We subsequently investigated the expression pattern and clinical significance of *ACTN1* and *ACTN4* in HNSCC using public datasets.

For *ACTN4*, our results indicated that its expression tends to either decrease or remain unchanged in tumor tissues compared to adjacent normal tissues (ANTs) or precancerous tissues across multiple HNSCC cohorts (Supplementary Fig. 1A-I). Furthermore, lower *ACTN4* expression was associated with significantly worse overall survival in HNSCC patients (Supplementary Fig. 1J-L), suggesting that ACTN4 may exert a tumor-suppressive role in HNSCC.

Importantly, ACTN4 protein expression remained largely unchanged in HNSCC cells following cisplatin exposure (Supplementary Fig. 1M-N), suggesting that it may not play a critical role in mediating cisplatin resistance in HNSCC cells. In contrast, *ACTN1* expression was significantly higher in HNSCC tissues than in ANTs (Supplementary Fig. 2A-E). As corroborated in Supplementary Fig. 2F-I, *ACTN1* was overexpressed in tumor tissues compared to normal tissues or oral precancerous lesions. Survival analysis from multiple independent cohorts indicated that higher *ACTN1* levels were associated with markedly worse overall survival in HNSCC patients (Supplementary Fig. 2J-L). Western blot analysis confirmed a persistent increase of ACTN1 in tumor tissues relative to ANTs (Supplementary Fig. 2M-N), suggesting a potential role for ACTN1 as a tumor promoter in HNSCC.

Given the consistent upregulation of *ACTN1* in cisplatin-resistant cell lines, we subsequently assessed its association with cisplatin resistance in HNSCC. Following treatment with a consistent cisplatin concentration over varying durations or varied cisplatin concentrations over a fixed duration, ACTN1 protein expression progressively increased in line with the concentration and duration of cisplatin exposure, suggesting a time- and dose-dependent relationship (Fig. 1C-F). Moreover, ACTN1 mRNA and protein expression were significantly higher in cisplatin-resistant cell lines than in their parental counterparts (Fig. 1G-H, Supplementary Fig. 2O). Furthermore, ACTN1 expression was notably higher in chemoresistant HNSCC tumors than in chemosensitive ones (Fig. 1I). Importantly, a progressive increase in ACTN1 expression was observed across tissues, corresponding to varying responses to neoadjuvant chemotherapy—ranging from complete response (CR), through partial response (PR) and stable disease (SD), to progressive disease (PD) (Fig. 1J-K). Therefore, increased ACTN1 expression in tissues prior to chemotherapy could potentially serve as a predictive biomarker for less favorable therapeutic responses. Stratifying our in-house chemotherapy-treated HNSCC cohort based on the median ACTN1 staining score revealed that high ACTN1 expression was associated with worse overall survival (Fig. 1L). Collectively, these findings suggest that ACTN1 may play a pivotal role in promoting tumorigenesis and enhancing cisplatin resistance in HNSCC.

As HPV-negative HNSCC is biologically and clinically distinct from HPV-positive HNSCC, we investigated the potential association of *ACTN1* expression with overall survival in both HPV-negative and -positive HNSCC. Among HPV-negative HNSCC patients from the TCGA database, high *ACTN1* expression was significantly associated with poorer overall survival (Supplementary Fig. 3A). However, in HPV-positive HNSCC patients, no significant difference in survival was observed between the high and low *ACTN1* expression groups. This may be due to ACTN1 overexpression being a more effective predictor of survival in HPV-negative HNSCC or the limited sample size of HPV-positive patients in the TCGA cohort. In the GSE65858 dataset, patients with higher *ACTN1* expression had worse overall survival in both HPV-negative and -positive HNSCC (Supplementary Fig. 3B-C). Thus, larger cohort studies are needed to determine the predictive value of ACTN1 overexpression in HPV-positive HNSCC.

Additionally, we have revealed an association between ACTN1 expression and immune features using the THInCR tool [29, 30]. ACTN1 expression correlates strongly with a range of immune features in HPV-negative HNSCC (Supplementary Table 3), underscoring a potentially unique interaction between ACTN1 expression and the immune landscape specifically in HPV-negative HNSCC cases.

### Depletion of ACTN1 suppresses malignant behaviors and enhances cisplatin sensitivity of HNSCC cells in vitro and in vivo

**Fig. 2:**
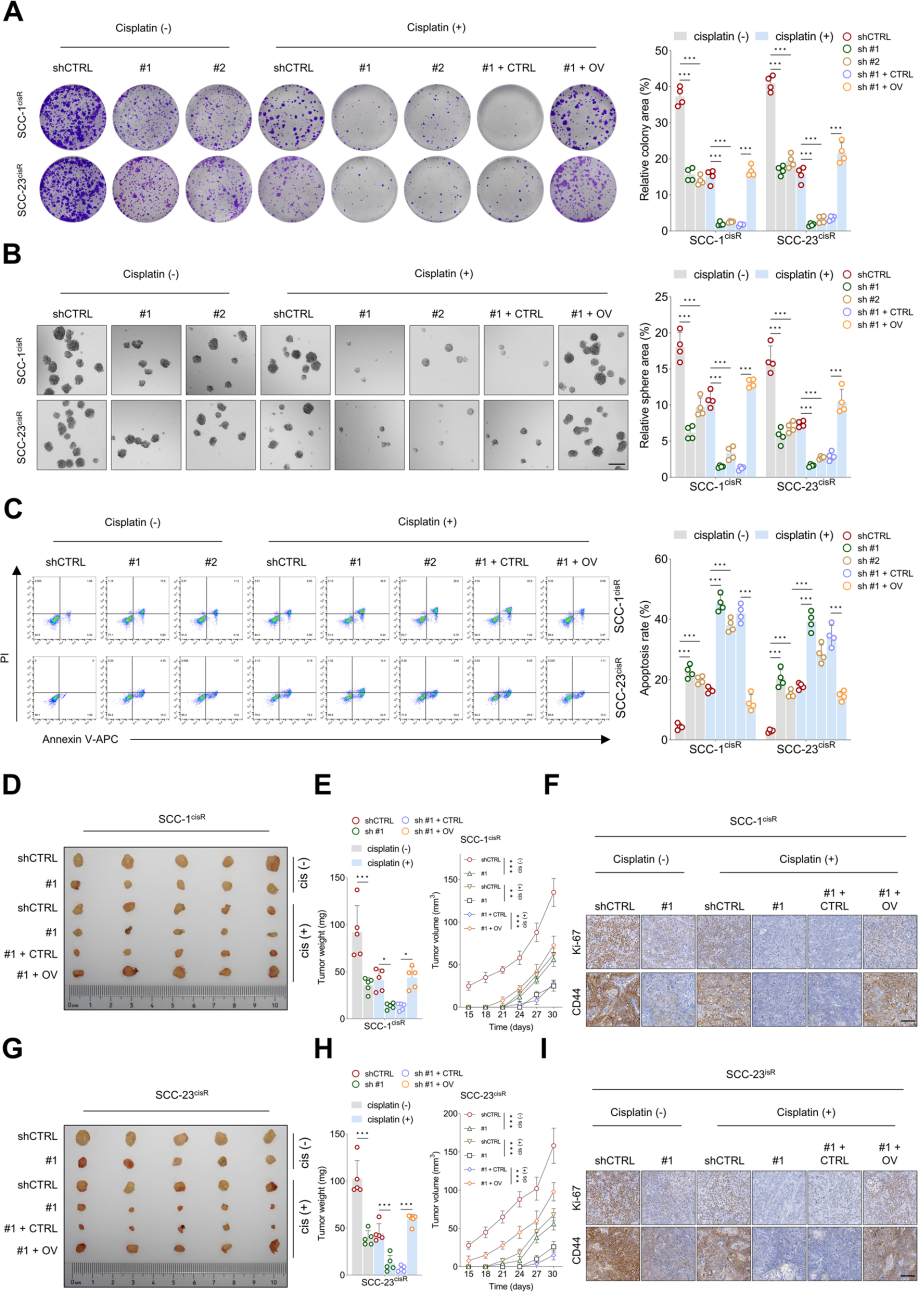
ACTN1 depletion suppresses malignancy and enhances cisplatin sensitivity of HNSCC cells both in vitro and in vivo. **A** Assessment of colony formation capacity in HNSCC cells subjected to designated modifications, with or without cisplatin treatment. **B** Analysis of the tumor sphere-forming ability in HNSCC cells, under specified modifications, and in the presence or absence of cisplatin. **C** Determination of the apoptosis rate in HNSCC cells following specified modifications, either treated with cisplatin or not. **D-F** Investigation of tumor size, weight, and volume in xenografts derived from SCC-1^cisR^ cells across different treatment groups. This is coupled with the examination of Ki-67 and CD44 staining intensities within corresponding tumor tissues. **G-I** Comparative evaluation of tumor size, weight, and volume in xenografts generated by SCC-23^cisR^ cells from specific treatment groups, together with the analysis of Ki-67 and CD44 staining intensities in associated tumor tissues. *P < 0.05, **P < 0.01, ***P < 0.001

We then investigated the effects of ACTN1 upregulation and downregulation on the malignancy and cisplatin resistance of HNSCC cells. Efficient modulation of ACTN1 expression in HNSCC cells was achieved using lentivirus-mediated systems (Supplementary Fig. 4A-B). MTT assay revealed a significant reduction in optical density values in the ACTN1-depleted groups, an effect which was more pronounced when combined with cisplatin treatment. Importantly, the overexpression of ACTN1 partially counterbalanced the reduced proliferative activity observed in HNSCC cells under this combined treatment (Supplementary Fig. 4C-D). Similar trends were observed in colony and sphere formation assays. ACTN1 depletion significantly curtailed the ability of HNSCC cells to form colonies and spheres, an effect that was further enhanced with the addition of cisplatin. Nevertheless, ACTN1 overexpression was able to partially restore these capabilities (Fig. 2A-B). In addition, apoptosis assay demonstrated that ACTN1 depletion, particularly when coupled with cisplatin treatment, induced a marked increase in HNSCC cell apoptosis, an effect partially mitigated by ACTN1 overexpression (Fig. 2C). In line with the in vitro findings, ACTN1 depletion in SCC-1^cisR^ and SCC-23^cisR^ cells led to a significant reduction in tumor size, volume, and weight in vivo. This effect was amplified by the concurrent administration of cisplatin. However, the overexpression of ACTN1 partially mitigated these reductions, demonstrating its influence on HNSCC progression and cisplatin resistance. Furthermore, IHC analysis indicated a marked decrease in the staining intensities of Ki-67 and CD44, markers of cellular proliferation and cancer stemness, respectively, under conditions of ACTN1 depletion. This effect was again enhanced by cisplatin treatment, but was partially counterbalanced by ACTN1 overexpression. These in vivo results, observed consistently in both SCC-1^cisR^ and SCC-23^cisR^ cells (Fig. 2D-I, Supplementary Fig. 4E-F), further corroborate our in vitro observations and emphasize the critical role of ACTN1 in HNSCC malignancy and cisplatin resistance.

### ACTN1 enhances oncogenic potential and drug resistance in HNSCC via activation of β-catenin-mediated pathway

**Fig. 3:**
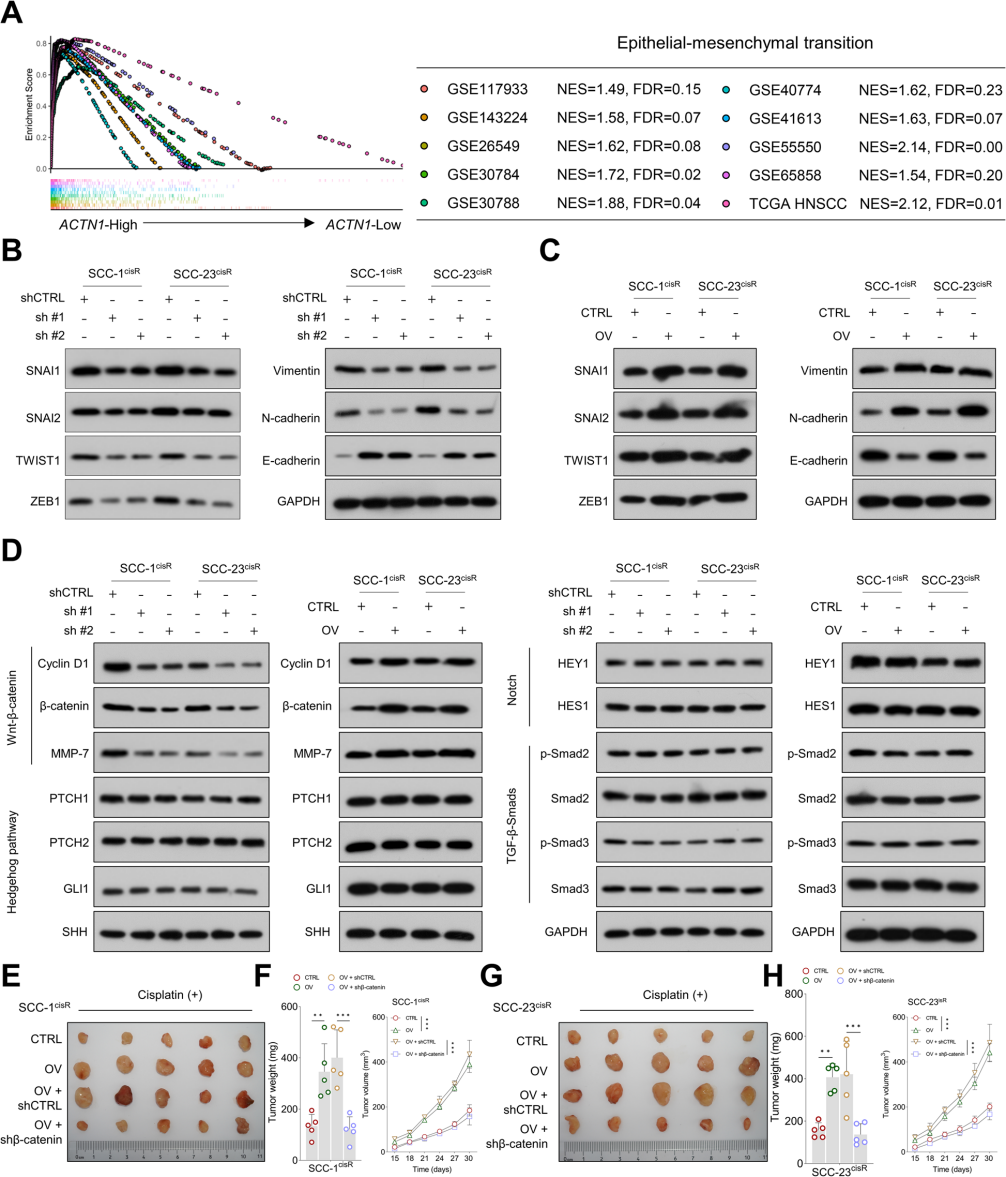
ACTN1 enhances tumorigenesis and cisplatin resistance in HNSCC cells via activation of β-catenin signaling. **A** Enrichment of the EMT pathway in the *ACTN1*-high group across multiple independent HNSCC cohorts, as indicated by GSEA analysis. **B** Western blotting analysis of SNAI1, SNAI2, TWIST1, ZEB1, Vimentin, N-cadherin, and E-cadherin expression in ACTN1-depleted cells and control cells. **C** Western blotting analysis of the effect of ACTN1 overexpression on EMT-associated marker levels in HNSCC cells. **D** Influence of ACTN1 depletion or overexpression on the levels of β-catenin-mediated signaling pathway components and the expression of key mediators in the Notch, Hedgehog, and TGF-β-Smad pathways. **E–F** Evaluation of tumor characteristics including size, weight, and volume in SCC-1^cisR^ cells following the indicated treatments. **G-H** Analysis of tumor size, weight, and volume in xenografts formed by SCC-23^cisR^ cells subjected to the specified treatments. **P < 0.01, ***P < 0.001

In order to unravel the potential molecular mechanisms responsible for ACTN1's role in the oncogenesis and drug resistance of HNSCC, we stratified HNSCC patients from the TCGA HNSCC cohort into *ACTN1*-high and *ACTN1*-low groups based on the median *ACTN1* expression value. GSEA revealed significant enrichment of the epithelial–mesenchymal transition (EMT) pathway in the *ACTN1*-high group, a finding that was corroborated across multiple independent cohorts (Fig. 3A). EMT process plays a crucial role in cancer progression, drug resistance, and increased stemness of tumors [31, 32]. Consistent with this role, our observations showed that ACTN1 depletion markedly reduced the expression of several EMT markers, including SNAI1, SNAI2, TWIST1, ZEB1, Vimentin, and N-cadherin. In contrast, E-cadherin expression, an epithelial marker, was significantly increased in cells in which ACTN1 was knocked down (Fig. 3B). Conversely, ACTN1 overexpression resulted in the opposite effect (Fig. 3C), further emphasizing ACTN1's central role in modulating the EMT process. The Wnt/β-catenin, Notch, Hh, and TGF-β/Smad signaling pathways are well-established regulators of the EMT process [33, 34]. Our data showed that ACTN1 depletion led to a decrease in the expression of cyclin D1, β-catenin, and MMP-7 (markers of the Wnt/β-catenin signaling pathway), with the opposite trends observed upon ACTN1 overexpression. However, ACTN1 overexpression or depletion had minimal impact on the protein levels of PTCH1, PTCH2, GLI1, and SHH (components of the Hh signaling pathway); HEY1 and HES1 (elements of the Notch signaling pathway); and Smad2, p-Smad2, Smad3, and pSmad3 (components of the TGF-β/Smad signaling pathway) (Fig. 3D). Moreover, ACTN1 expression positively correlated with the expression of β-catenin target genes across various independent HNSCC cohorts (Supplementary Fig. 5A-D), and ACTN1 depletion significantly decreased the levels of β-catenin target genes (Supplementary Fig. 5E-F). TOPFlash luciferase activity was found to be markedly reduced in ACTN1-depleted cells, whereas its overexpression led to a noticeable increase in the activity (Supplementary Fig. 6A-B). Moreover, ACTN1 overexpression boosted the colony- and sphere-forming capacities of HNSCC cells, and β-catenin knockdown almost entirely negated the oncogenic effects of ACTN1 overexpression (Supplementary Fig. 6C-F). Importantly, in vivo studies further confirmed that tumor growth driven by ACTN1 upregulation was halted by β-catenin depletion (Fig. 3E-H). Collectively, these findings strengthen the hypothesis that ACTN1 promotes HNSCC malignancy via activation of the β-catenin-mediated pathway.

### ACTN1 partially activates β-catenin signaling via the integrin β1-FAK-PI3K-AKT pathway in HNSCC

**Fig. 4:**
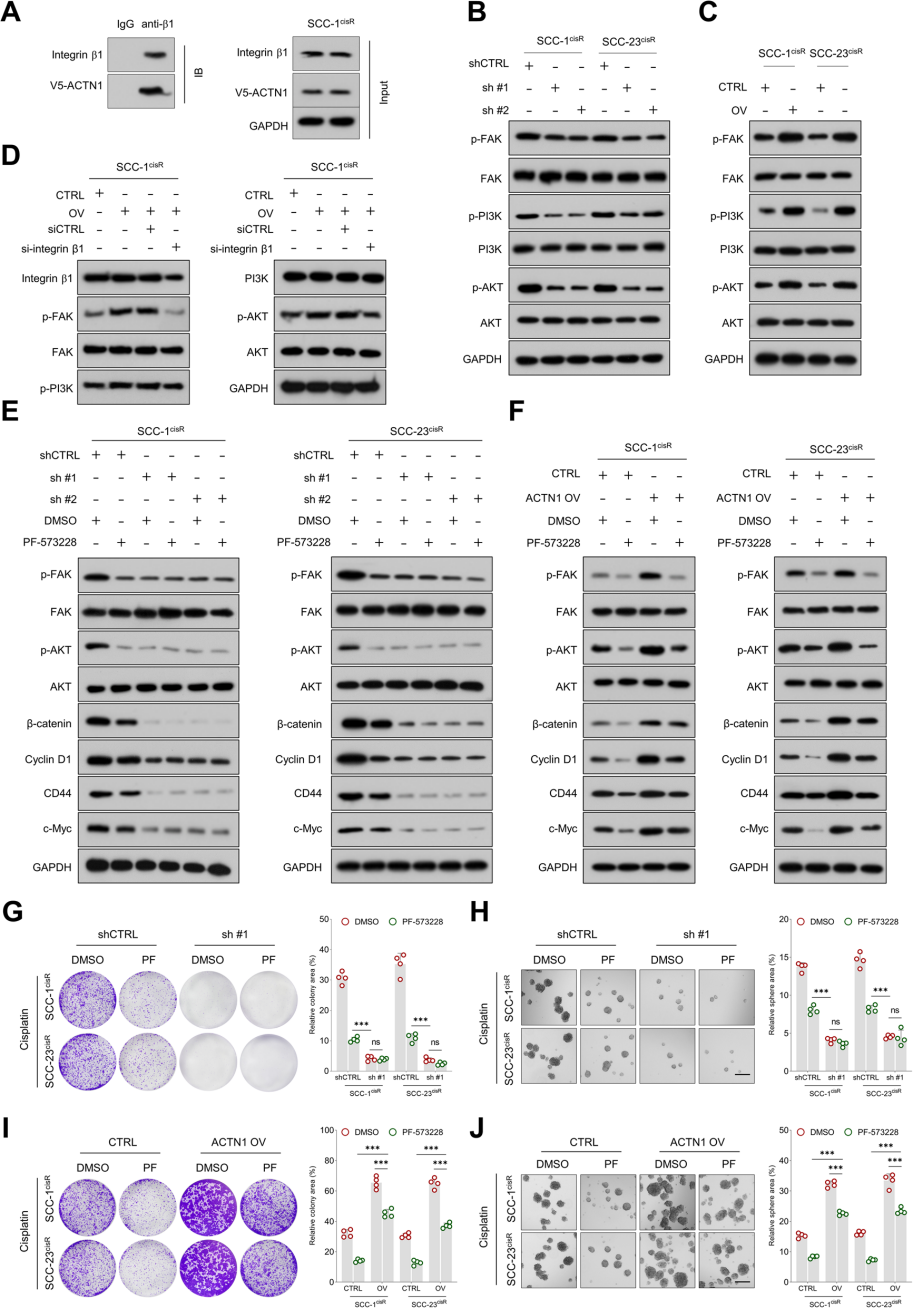
ACTN1 mediates activation of β-catenin signaling via the integrin β1-FAK-PI3K-AKT pathway. **A** Analysis of the interaction between ACTN1 and integrin β1 in SCC-1^cisR^ cells. **B-C** Influence of ACTN1 depletion or overexpression on phosphorylation and total levels of FAK, PI3K, and AKT. **D** Western blotting analysis of the effects of integrin β1 depletion on p-FAK, FAK, p-PI3K, PI3K, p-AKT, and AKT expression in ACTN1-overexpressing SCC-1^cisR^ cells. **E** Assessment of β-catenin and its downstream targets' levels in ACTN1-depleted cells and their controls, treated with or without PF-573228. **F** Analysis of β-catenin and its downstream targets' expression in control cells and ACTN1-overexpressing cells, with or without PF-573228 administration. **G-H** Evaluation of the colony-forming potential of HNSCC cells in control and ACTN1-depleted groups, with or without PF-573228 treatment. **I-J** Assessment of the sphere-forming capacity of HNSCC cells in control and ACTN1-overexpressing groups, with or without PF-573228 treatment. ***P < 0.001, ns: not significant

Previous study has identified a physical interaction between ACTN1 and integrin β1 in fibroblasts [35]. This finding is supported by our bioinformatics analysis, which revealed significant enrichment of the integrin 1 pathway in the *ACTN1*-high group across multiple independent HNSCC cohorts (Supplementary Fig. 7A). Co-IP assays further validated this interaction in HNSCC cells (Fig. 4A, Supplementary Fig. 7B). Considering the well-established role of integrin β1 in activating FAK, leading to subsequent phosphorylation of PI3K and AKT and the activation of the Wnt/β-catenin pathway [36, 37], we hypothesized that ACTN1 might regulate β-catenin signaling through the integrin β1-FAK-PI3K-AKT axis. The FAK pathway was also found to be enriched in the *ACTN1*-high group in multiple independent HNSCC cohorts (Supplementary Fig. 7C). Supporting our hypothesis, ACTN1 depletion led to decreased levels of p-FAK, p-PI3K, and p-AKT, while ACTN1 overexpression increased these phosphorylated proteins (Fig. 4B-C). Furthermore, knockdown of integrin β1 neutralized the ACTN1 overexpression effect on the levels of p-FAK, p-PI3K, and p-AKT (Fig. 4D, Supplementary Fig. 8A).

When we evaluated the role of the FAK-PI3K-AKT signaling network in ACTN1-mediated β-catenin pathway activity, the expression levels of β-catenin, its downstream targets, and TOPFlash luciferase activity were unexpectedly higher in the control group undergoing FAK inhibition compared to the ACTN1-depleted group, regardless of the presence of PF-573228. Additionally, the effects of ACTN1 upregulation on β-catenin-mediated signaling were only partially mitigated by the addition of PF-573228 (Fig. 4E-F, Supplementary Fig. 8B-E). Intriguingly, both colony-forming and sphere-forming abilities of HNSCC cells were significantly higher following FAK inhibition than after ACTN1 depletion, indicating that FAK suppression only partially repressed the malignant behaviors of HNSCC cells (Fig. 4G-H). Likewise, FAK inhibition only partially reduced the enhancements of colony- and sphere-forming abilities induced by ACTN1 overexpression in HNSCC cells (Fig. 4I-J). This suggests that, in addition to the FAK-PI3K-AKT pathway, other important molecular mechanisms could be involved in the regulation of the β-catenin pathway by ACTN1.

### ACTN1 promotes GSK-3β degradation by enhancing its interaction with MYH9

**Fig. 5:**
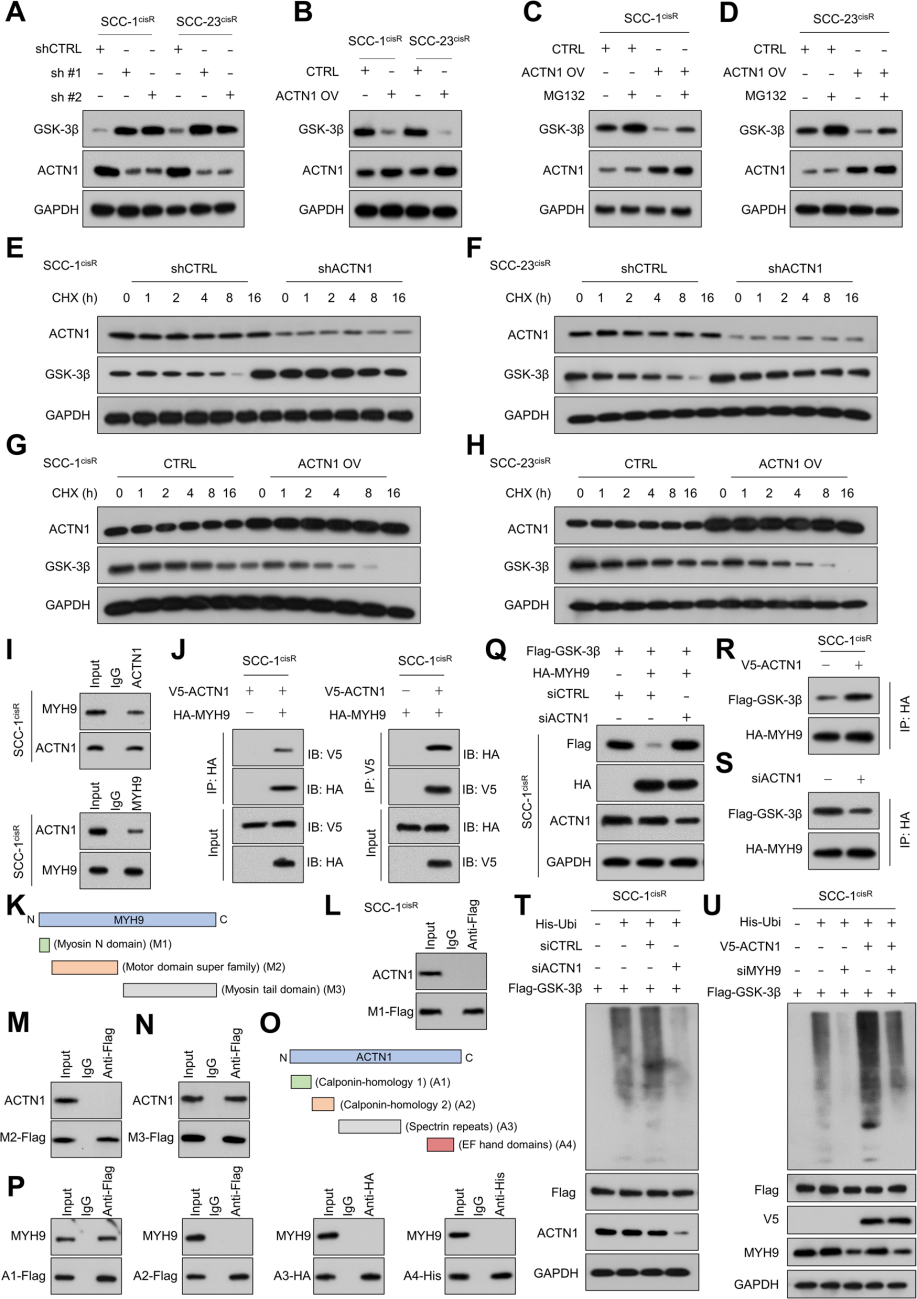
ACTN1 promotes GSK-3β degradation by enhancing its interaction with MYH9. **A** GSK-3β expression in ACTN1-depleted cells and control cells. **B** GSK-3β levels in ACTN1-overexpressing cells and control cells. **C-D** Influence of ACTN1 upregulation on GSK-3β expression in HNSCC cells with or without the proteasome inhibitor, MG132. **E–F** Degradation rate of GSK-3β in ACTN1-depleted HNSCC cells compared to control cells. **G-H** Effect of ACTN1 overexpression on the half-life of GSK-3β in HNSCC cells. **I-J** Interaction between ACTN1 and MYH9 in HNSCC cells as demonstrated by endogenous and exogenous Co-IP assays. **K** Schematic representation of MYH9 domains. **L-N** Interactions between MYH9 domains and ACTN1 in SCC-1^cisR^ cells as revealed by Co-IP experiments. **O** Schematic representation of ACTN1 domains. **P** Interactions between ACTN1 domains and MYH9 in SCC-1 cells as illustrated by Co-IP experiments. **Q** GSK-3β expression in HNSCC cells subjected to indicated treatments. **R-S** Effects of ACTN1 overexpression or depletion on the interaction between MYH9 and GSK-3β in HNSCC cells. **T** Impact of ACTN1 depletion on the ubiquitination level of GSK-3β in HNSCC cells. **U** Ubiquitination level of GSK-3β in HNSCC cells subjected to specified modifications

GSK-3β, a serine/threonine kinase, plays a crucial role in negatively regulating the oncogenic Wnt/β-catenin signaling pathway [38]. Initially, we examined the impact of ACTN1 on GSK-3β expression. Western blot analysis showed that ACTN1 depletion significantly increased GSK-3β expression, whereas ACTN1 overexpression led to a marked reduction in GSK-3β levels (Fig. 5A-B). Interestingly, ACTN1's inhibitory effect on GSK-3β expression was partially reversed when treated with the proteasome inhibitor MG132 (Fig. 5C-D), suggesting that ACTN1 may decrease GSK-3β stability through a proteasome-dependent degradation pathway. This mechanism was further supported by a CHX chase assay, which showed an increase in the half-life of GSK-3β when ACTN1 was downregulated, while its upregulation led to a decrease in GSK-3β's half-life (Fig. 5E-H, Supplementary Fig. 9A-D). Subsequently, we performed an IP assay followed by MS to identify proteins interacting with ACTN1. MYH9, a known promoter of GSK-3β degradation [39], was identified as the top candidate (Supplementary Table 4). Therefore, we hypothesized that ACTN1 might augment GSK-3β degradation by fostering the interaction between MYH9 and GSK-3β. Supporting this, both endogenous and exogenous Co-IP experiments revealed a physical interaction between ACTN1 and MYH9 (Fig. 5I-J, Supplementary Fig. 9E-F). We also determined that the calponin-homology 1 domain of ACTN1 and the myosin tail domain of MYH9 were the respective interaction sites (Fig. 5K-P). In line with these observations, MYH9 overexpression resulted in a significant reduction in GSK-3β expression in HNSCC cells, an effect that was largely counteracted by ACTN1 depletion (Fig. 5Q, Supplementary Fig. 9G). Additionally, ACTN1 depletion decreased the binding of MYH9 to GSK-3β, while enforced ACTN1 expression enhanced this interaction (Fig. 5R-S, Supplementary Fig. 9H-I). These findings strongly suggest that ACTN1 promotes the interaction between MYH9 and GSK-3β, facilitating the degradation of GSK-3β and leading to subsequent β-catenin activation. Finally, we assessed the effects of ACTN1 and MYH9 on GSK-3β ubiquitination. As predicted, GSK-3β ubiquitination decreased following ACTN1 depletion, whereas ACTN1 overexpression led to enhanced GSK-3β polyubiquitination. Importantly, downregulation of MYH9 significantly reduced GSK-3β polyubiquitination, an effect that was partially mitigated by coexpression of ACTN1 (Fig. 5T-U, Supplementary Fig. 9J-K).

### MYH9 depletion mitigates the tumor-promoting impact of ACTN1 overexpression in HNSCC

**Fig. 6:**
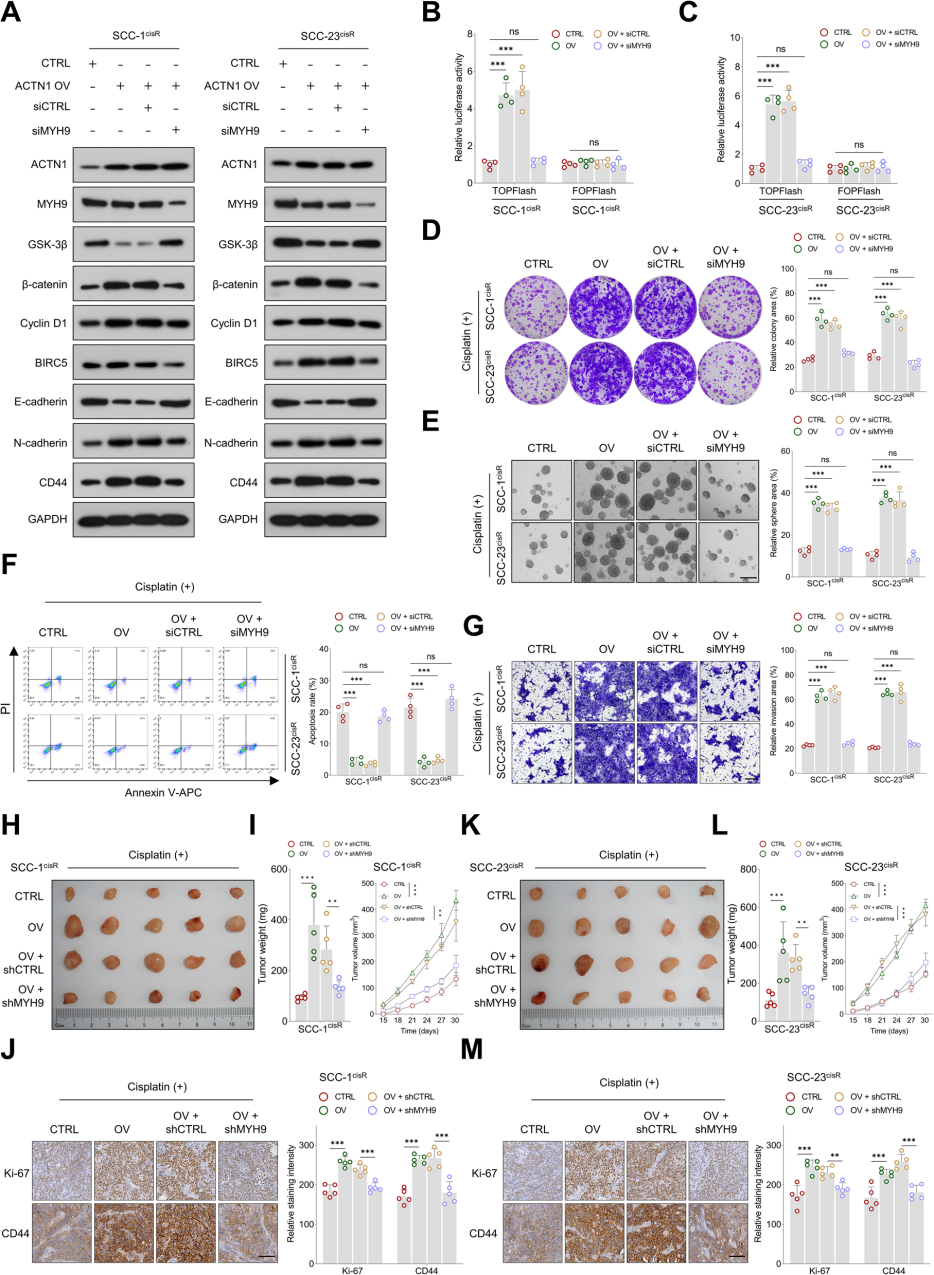
MYH9 depletion mitigates the tumor promoting effects of ACTN1 overexpression in HNSCC. **A** Western blotting analyses of ACTN1, MYH9, GSK-3β, β-catenin, cyclin D1, BIRC5, E-cadherin, N-cadherin, and CD44 expression in HNSCC cells following indicated treatments. **B-C** Assessment of relative TOPFlash and FOPFlash activities in HNSCC cells subjected to the indicated modifications. **D-E** Evaluation of colony-forming and sphere-forming capacities of HNSCC cells following the indicated treatments. **F** Analysis of the apoptosis rate in HNSCC cells subjected to the indicated treatments. **G** Assessment of the invasive capabilities of HNSCC cells following the indicated treatments. **H-J** Analysis of tumor size, weight, and volume in xenografts generated by SCC-1^cisR^ cells from specified treatment groups, and examination of the staining intensities of Ki-67 and CD44 in corresponding tumor tissues. **K-M** Evaluation of tumor size, weight, and volume in xenografts formed by SCC-23^cisR^ cells from indicated treatment groups, and examination of the staining intensities of Ki-67 and CD44 in the respective tumor tissues. **P < 0.01, ***P < 0.001, ns: not significant

We then evaluated whether MYH9 depletion could counteract the tumor-promoting role of ACTN1 in HNSCC. Western blotting revealed that ACTN1 overexpression resulted in elevated levels of β-catenin, cyclin D1, BIRC5, N-cadherin, and CD44, while simultaneously suppressing GSK-3β and E-cadherin expression. These effects were negated by MYH9 knockdown (Fig. 6A).

Moreover, TOPFlash luciferase activity, which was increased by ACTN1 overexpression, was diminished by MYH9 depletion (Fig. 6B-C). Ectopic ACTN1 expression significantly enhanced the colony-forming, sphere-forming, and invasive abilities of HNSCC cells, and also decreased their apoptosis. These tumor-promoting effects of ACTN1 overexpression were largely counteracted by MYH9 downregulation (Fig. 6D-G). Our in vivo experiments further provided support for these findings, as MYH9 depletion in SCC-1^cisR^ cells notably decreased the tumor size, weight, and volume, which had all been increased by ACTN1 overexpression (Fig. 6H-I). Additionally, the intensities of Ki67 and CD44 staining, enhanced by ACTN1 upregulation, were markedly reduced by MYH9 downregulation in xenograft tumor tissues (Fig. 6J). Comparable results were observed in SCC-23^cisR^ cells (Fig. 6K-M), further substantiating these findings.

### β-catenin-c-Myc axis forms a positive feedback loop for upregulating ACTN1 in HNSCC

**Fig. 7:**
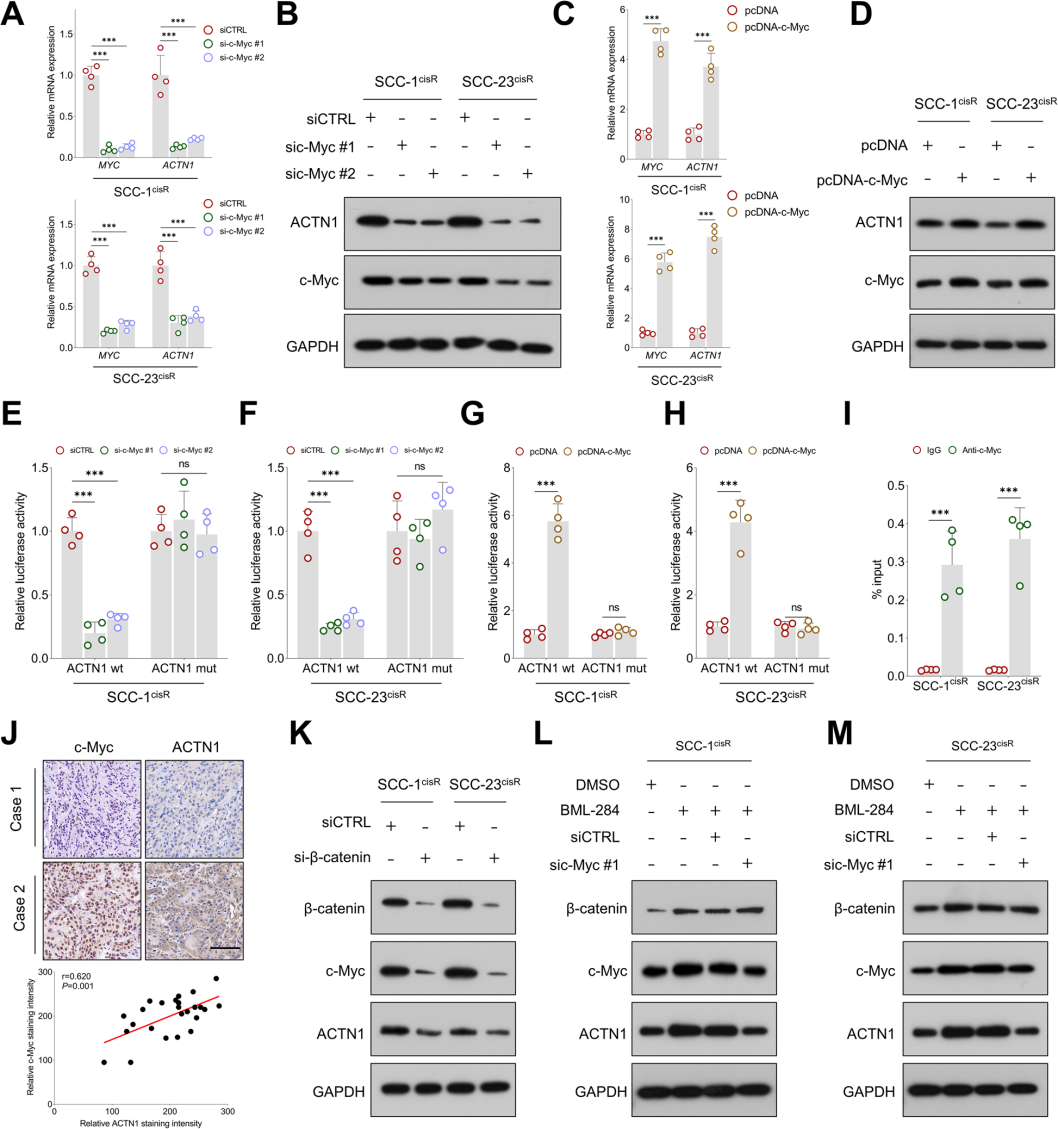
β-catenin/c-Myc axis forms a positive feedback loop for enhancing ACTN1 expression in HNSCC. **A-B** qPCR and western blotting analyses assessing c-Myc and ACTN1 expression in HNSCC cells following siRNA-mediated c-Myc knockdown. **C-D** qPCR and western blotting analyses of c-Myc and ACTN1 expression in HNSCC cells with c-Myc overexpression. **E–F** Examination of the effects of c-Myc depletion on the luciferase activities in HNSCC cells transfected with either the wild-type or mutant-type ACTN1 promoter. **G-H** Assessment of the influence of c-Myc overexpression on luciferase activities in HNSCC cells transfected with either the wild-type or mutant-type ACTN1 promoter. **I** Enrichment of c-Myc in the ACTN1 promoter region, as revealed by ChIP-qPCR. **J** Correlation analysis of c-Myc and ACTN1 staining intensities in HNSCC tissues from the in-house cohort. **K** Western blotting analysis of c-Myc and ACTN1 expression levels in HNSCC cells after β-catenin depletion. **L-M** Western blotting analysis of c-Myc and ACTN1 expression levels in HNSCC cells treated with the β-catenin signaling activator, BML-284, with or without sic-Myc. ***P < 0.001, ns: not significant

c-Myc was identified as a potential upstream transcriptional factor for ACTN1 based on bioinformatics analysis (c-Myc binding site: GTCACATG). Consistent with this, c-Myc knockdown reduced ACTN1 expression at both mRNA and protein levels (Fig. 7A-B), while overexpression of c-Myc produced the opposite effect (Fig. 7C-D).

Moreover, a luciferase reporter assay revealed that c-Myc positively regulated the luciferase activity of the wild-type ACTN1 promoter but had minimal impact on the mutant-type ACTN1 promoter activity (Fig. 7E-H). ChIP-qPCR further demonstrated that c-Myc was enriched in the promoter region of ACTN1 (Fig. 7I). These observations suggest that c-Myc transcriptionally regulates ACTN1 expression in HNSCC cells. Interestingly, a strong positive correlation was observed between ACTN1 and c-Myc expression in our in-house cohort (Fig. 7J). Since c-Myc is a well-established downstream target of the β-catenin signaling pathway, we further investigated whether the β-catenin-c-Myc axis plays a role in regulating ACTN1 expression. Western blot analysis showed that β-catenin inhibition led to a reduction in the expression of both c-Myc and ACTN1 in HNSCC cells (Fig. 7K). Furthermore, depletion of c-Myc markedly attenuated the stimulatory effects of β-catenin activation on ACTN1 expression (Fig. 7L-M). This suggests that the β-catenin-c-Myc axis forms a positive feedback loop for upregulating ACTN1 in HNSCC.

### Targeting ACTN1 effectively overcomes cisplatin resistance in a HNSCC PDX model

**Fig. 8:**
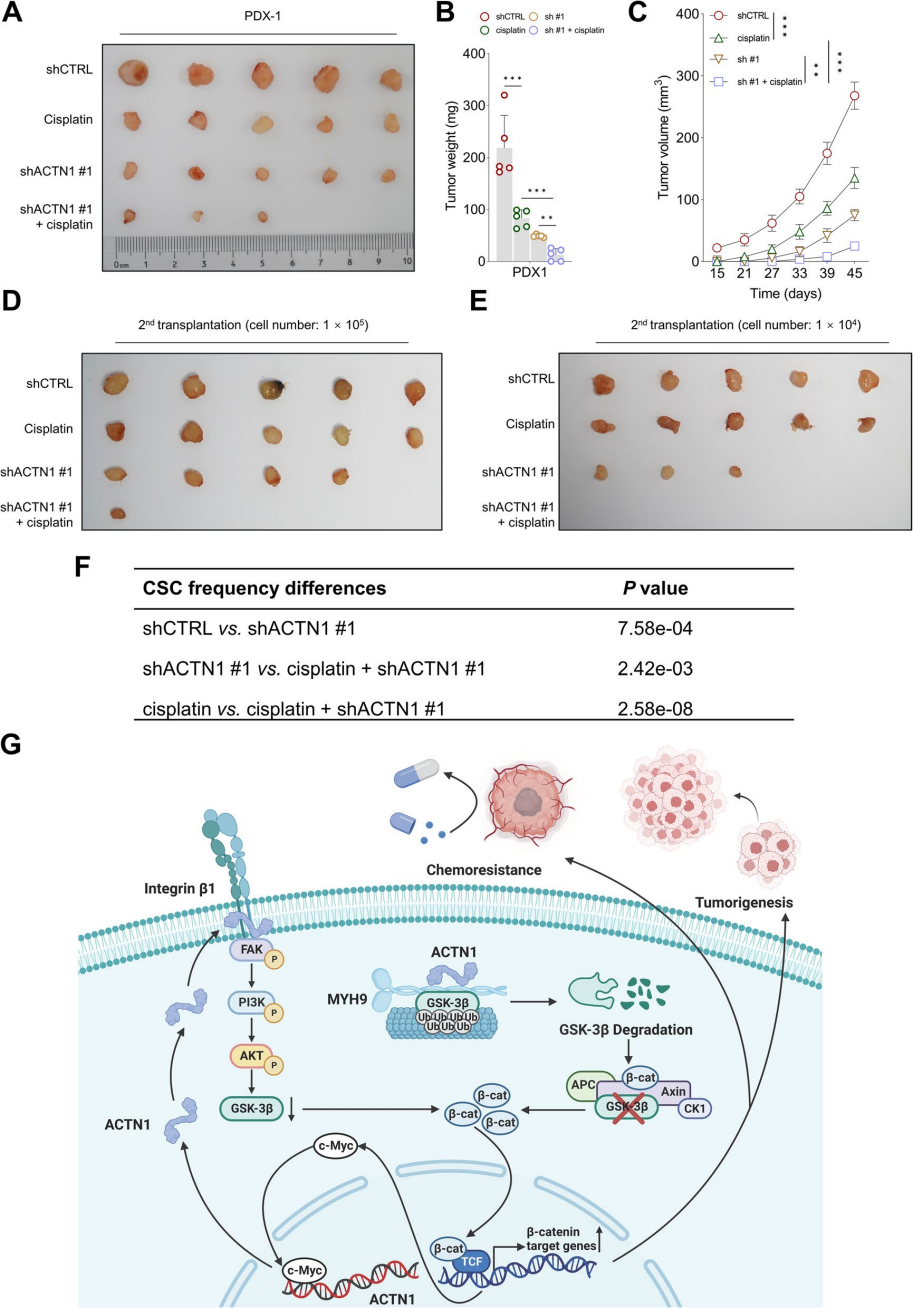
The synergistic antitumor effects of targeting ACTN1 and cisplatin in a PDX model. **A-C** Evaluation of tumor growth in PDX cells treated with a combination of ACTN1 inhibition and cisplatin, compared with cells subjected to either ACTN1 inhibition or cisplatin alone. **D-F** Assessment of tumor-initiating potential in cancer cells extracted from PDX tumors across the different treatment groups. **G** Schematic representation of the proposed molecular mechanisms, illustrating the dual role of ACTN1 in promoting tumorigenesis and conferring drug resistance in HNSCC. **P < 0.01, ***P < 0.001

The combined therapeutic potential of ACTN1 targeting and cisplatin treatment was further evaluated using a PDX model. Expression levels of β-catenin and its downstream targets were significantly reduced, while GSK-3β levels were noticeably increased in PDX-derived tumor cells subjected to ACTN1 depletion (Supplementary Fig. 10A-C). The PDX model demonstrated that ACTN1 depletion and cisplatin treatment had a synergistic antitumor effect (Fig. 8A-C).

Additionally, we evaluated the ability of the tumor to repopulate post-therapeutic interventions. Notably, the potential to generate secondary PDXs was significantly reduced in tumor cells treated with a combination of ACTN1 inhibition and cisplatin, compared to the robust tumorigenicity exhibited by cancer cells receiving other individual treatments (Fig. 8D-F, Supplementary Table 5). These findings highlight the efficacy of ACTN1 targeting as a promising strategy to overcome cisplatin resistance in HNSCC.

## Discussion

Our findings underscore the central role of ACTN1 in mediating HNSCC cisplatin resistance. Higher ACTN1 levels linked with poor prognosis and therapeutic responses highlight its clinical relevance. As illustrated in Fig. 8G, we further unraveled the multifaceted mechanisms through which ACTN1 orchestrates cisplatin resistance. Specifically, ACTN1 was found to instigate the ubiquitin-dependent degradation of GSK-3β by fostering an interaction with MYH9, which in turn activates the β-catenin pathway. Concurrently, ACTN1 was also demonstrated to engage with integrin β1, activating the FAK/PI3K/AKT pathway, subsequently leading to β-catenin stabilization. Intriguingly, ACTN1 expression itself was under the regulation of the β-catenin-c-Myc axis, constituting a self-reinforcing loop. These dual mechanistic pathways highlight the critical role of ACTN1 in modulating the cellular response to cisplatin in HNSCC, substantiating its promise as a compelling target for therapeutic intervention.

Chemoresistance remains a formidable obstacle in HNSCC treatment. The activation of β-catenin signaling, a well-documented player in the tumorigenesis and drug resistance of various cancers, including HNSCC, contributes to detrimental clinical outcomes [16, 40]. The Wnt/β-catenin pathway, for instance, orchestrates cellular processes that enable HNSCC cells to retain a stem-like phenotype and develop aggressive characteristics [41]. Moreover, aberrant β-catenin signaling activation promotes cisplatin resistance in HNSCC [42], leading to therapeutic failure and disease progression. Our findings position ACTN1 as a pivotal modulator of β-catenin signaling in HNSCC cells, in alignment with our anticipation of ACTN1 as a central mediator of cisplatin resistance in HNSCC. Crucially, we revealed a synergistic effect between ACTN1 depletion and cisplatin treatment in diminishing tumor growth, both in a xenograft and a PDX model. This underscores the potential effectiveness of ACTN1 targeting in overcoming resistant HNSCC. Moreover, ACTN1 may serve as a valuable predictive biomarker for chemotherapy responses and prognosis in HNSCC. Despite these findings, the lack of a specific small molecule drug targeting ACTN1 represents a significant challenge and highlights the urgency for the development of novel therapeutics aimed at depleting ACTN1 in HNSCC cells.

ACTN1 consists of an N-terminal actin-binding domain, a central rod domain, and a c-terminal calcium-binding domain [7]. In nonmuscle cells, ACTN1 localization at zonula adherens and focal adhesions indicates its involvement in cell communication and the formation of focal adhesions [9]. Our data reveal a physical interaction between ACTN1 and integrin β1, which consequently promotes FAK phosphorylation. We propose that ACTN1 may influence the physical interactions between cancer cells and their extracellular matrix, which could modulate integrin β1 activation. This process could foster the recruitment and ensuing phosphorylation of FAK, a fundamental event in integrin-mediated signal transduction. FAK, a cytoplasmic protein tyrosine kinase, plays a critical role in facilitating cell motility, survival, and proliferation in the tumor microenvironment [43]. Its regulation of cell signaling operates through both kinase-dependent and kinase-independent pathways. Our observations suggest that ACTN1 modulates β-catenin-mediated signaling primarily through the kinase-dependent PI3K/AKT pathway. Importantly, AKT serves as a key kinase regulator of GSK-3β, inhibiting GSK-3β activity via Ser9 phosphorylation [44]. Intriguingly, the inhibition of AKT does not entirely eliminate the tumor-promoting effects and the upregulated β-catenin-mediated signaling resulting from ACTN1 overexpression, suggesting the possibility of additional underlying molecular mechanisms.

β-catenin signaling is primarily regulated through the GSK-3β-dependent pathway, a well-established mechanism [38]. In our study, we delved into the effects of ACTN1 overexpression on GSK-3β, revealing that ACTN1 negatively impacts GSK-3β stability in HNSCC cells and expedites its degradation via the ubiquitin–proteasome pathway. MYH9, a nonmuscle myosin heavy chain IIA, is integral to cytokinesis, cell motility, and morphology. It has been associated with various diseases, including cancer, due to its aberrant expression patterns [45–47]. MYH9, known to enhance GSK-3β ubiquitination [39], physically interacts with ACTN1. This interaction facilitates the binding of MYH9 to GSK-3β, promoting its ubiquitination and subsequent degradation. We further demonstrated that the calponin-homology 1 domain of ACTN1 physically binds to the myosin tail domain of MYH9, suggesting a potential role for these domains in facilitating MYH9-mediated GSK-3β ubiquitination. In summary, our data suggest that ACTN1-associated MYH9 enhances GSK-3β ubiquitination and promotes its degradation. Notably, silencing MYH9 significantly attenuates ACTN1's tumor-promoting effects, supporting the notion that ACTN1 drives HNSCC tumorigenesis primarily by boosting MYH9-dependent GSK-3β degradation. Moreover, we discovered that ACTN1 not only augments the β-catenin signaling pathway but is also transcriptionally regulated by the β-catenin/c-Myc axis. This reciprocal relationship forms a self-amplifying feedback loop, perpetuating the malignant phenotype of HNSCC cells. Unveiling this self-sustaining circuit of ACTN1 expression and the intertwined regulation with the β-catenin/c-Myc axis sheds light on a fundamental mechanism that underlies the aggressive and resistant traits of HNSCC. This revelation underscores the β-catenin/c-Myc/ACTN1 axis as a promising therapeutic target for mitigating the pathogenesis of HNSCC.

Several limitations of our study should be noted. First, in our in vivo experiments, we employed BALB/c nude mice, which inherently lack a functional adaptive immune system. This may lead to a lack of insight into the complex intricacies of the tumor microenvironment (TME) and the dynamic interactions with anti-tumor immunity. Cisplatin is known to modulate various TME components, such as innate immunity, tumor vascularization, cancer-associated fibroblasts (CAFs), and hypoxic conditions. For instance, cisplatin administration has been shown to decrease both the number and function of regulatory T cells in lung tumor mouse model [48]. Furthermore, cisplatin at moderate doses boosts antitumor immunity and, when paired with anti–PD-L1/PD-1 therapy, improves survival in HNSCC without additional toxicity [49]. The complex interplay between cisplatin and the TME affects not only the direct anti-tumor responses but also the modulation of drug resistance. Therefore, further exploration into the intricate crosstalk between cisplatin and the TME is essential. Second, the impact of ACTN1 targeting on the TME and tumor immunity in HNSCC necessitates further investigation. Third, the urgent development of small molecules that specifically target ACTN1 is also needed.

In conclusion, our study highlights the aberrant overexpression of ACTN1 in HNSCC, correlating this heightened expression to cisplatin resistance and unfavorable patient prognoses. The underlying mechanisms through which ACTN1 fosters HNSCC drug resistance and tumorigenesis encompass the activation of β-catenin via both MYH9-dependent GSK-3β degradation and intergrin β1-mediated FAK phosphorylation. The critical role of ACTN1 in modulating chemosensitivity in HNSCC suggests its potential as a robust predictive biomarker for chemotherapy responses and as a candidate for therapeutic intervention to overcome drug resistance.

### Supplementary Information

**Additional file 1: Supplementary Fig. S1.** Characterization of ACTN4 expression profile and its clinical significance in HNSCC. **Supplementary Fig. S2.** Investigation of ACTN1 expression patterns and its clinical significance in HNSCC. **Supplementary Fig. S3.** Association of ACTN1 expression with overall survival in HPV-negative and positive HNSCC. **Supplementary Fig. S4.** Impact of ACTN1 depletion on malignant phenotypes and cisplatin sensitivity of HNSCC cells in vitro and in vivo. **Supplementary Fig. S5.** Role of ACTN1 in β-catenin signaling activation in HNSCC cells. **Supplementary Fig. S6.** ACTN1 promotes the oncogenic behaviors of HNSCC cells through activating β-catenin signaling. **Supplementary Fig. S7.** Enrichment of Integrin β1 and FAK pathways in the ACTN1-high group across multiple independent HNSCC cohorts. **Supplementary Fig. S8.** Partial mitigation of ACTN1 overexpression-enhanced β-catenin signaling through FAK inhibition. **Supplementary Fig. S9.** Promotion of GSK-3β degradation by ACTN1 via enhanced interaction with MYH9. **Supplementary Fig. S10.** Suppression of β-catenin-mediated signaling in tumor cells from PDX tumors by ACTN1 depletion. **Supplementary Table S1.** The clinicopathological information of the HNSCC patients. **Supplementary Table S2.** Sequences of primers and oligos used in this study. **Supplementary Table 3.** The correlation of ACTN1 mRNA expression and immune function in HPV (-) and HPV (+) HNSCC patients. **Supplementary Table S4.** The proteins interacting with ACTN1 identified by mass spectrometry. **Supplementary Table S5.** The tumor-initiating capacity of cancer cells isolated from PDXs with indicated modifications.

## Data Availability

The data that support the findings of this study are available from the corresponding authors upon reasonable request.

## Abbreviations

ACTN1: Alpha-actinin 1
ANTs: Adjacent normal tissues
CR: Complete response
CSCs: Cancer stem cells
EMT: Epithelial–mesenchymal transition
HNSCC: Head and neck squamous cell carcinoma
MYH9: Myosin heavy chain 9
PD: Progressive disease
PR: Partial response
SD: Stable disease
