# Supplementary material for: ACTN1 promotes HNSCC tumorigenesis and cisplatin resistance by enhancing MYH9-dependent degradation of GSK-3β and integrin β1-mediated phosphorylation of FAK
Source: J Exp Clin Cancer Res. 2023 Dec 7;42:335. doi: 10.1186/s13046-023-02904-w (PMC10701957; doi:10.1186/s13046-023-02904-w)
Supplement: Supplementary file 1 — Additional file 1: Supplementary Fig. S1. Characterization of ACTN4 expression profile and its clinical significance in HNSCC. Supplementary Fig. S2. Investigation of ACTN1 expression patterns and its clinical significance in HNSCC. Supplementary Fig. S3. Association of ACTN1 expression with overall survival in HPV-negative and positive HNSCC. Supplementary Fig. S4. Impact of ACTN1 depletion on malignant phenotypes and cisplatin sensitivity of HNSCC cells in vitro and in vivo. Supplementary Fig. S5. Role of ACTN1 in β-catenin signaling activation in HNSCC cells. Supplementary Fig. S6. ACTN1 promotes the oncogenic behaviors of HNSCC cells through activating β-catenin signaling. Supplementary Fig. S7. Enrichment of Integrin β1 and FAK pathways in the ACTN1-high group across multiple independent HNSCC cohorts. Supplementary Fig. S8. Partial mitigation of ACTN1 overexpression-enhanced β-catenin signaling through FAK inhibition. Supplementary Fig. S9. Promotion of GSK-3β degradation by ACTN1 via enhanced interaction with MYH9. Supplementary Fig. S10. Suppression of β-catenin-mediated signaling in tumor cells from PDX tumors by ACTN1 depletion. Supplementary Table S1. The clinicopathological information of the HNSCC patients. Supplementary Table S2. Sequences of primers and oligos used in this study. Supplementary Table 3. The correlation of ACTN1 mRNA expression and immune function in HPV (-) and HPV (+) HNSCC patients. Supplementary Table S4. The proteins interacting with ACTN1 identified by mass spectrometry. Supplementary Table S5. The tumor-initiating capacity of cancer cells isolated from PDXs with indicated modifications. [file 13046_2023_2904_MOESM1_ESM.pdf]

Supplementary material for

ACTN1 promotes HNSCC tumorigenesis and cisplatin resistance by enhancing MYH9-dependent degradation of GSK-3β and integrin β1-mediated phosphorylation of FAK

Li Cui[1#], Ye Lu[1], Jiarong Zheng[2], Bing Guo[2], Xinyuan Zhao[1#]

[1]Stomatological Hospital, School of Stomatology, Southern Medical University, Guangzhou, 510280, Guangdong, China.

[2]Department of Dentistry, the First Affiliated Hospital, Sun Yat-Sen University, Guangzhou 510080, China.

#Correspondence

Li Cui, Email: licui@smu.edu.cn

Xinyuan Zhao, Email: zhaoxinyuan1989@smu.edu.cn

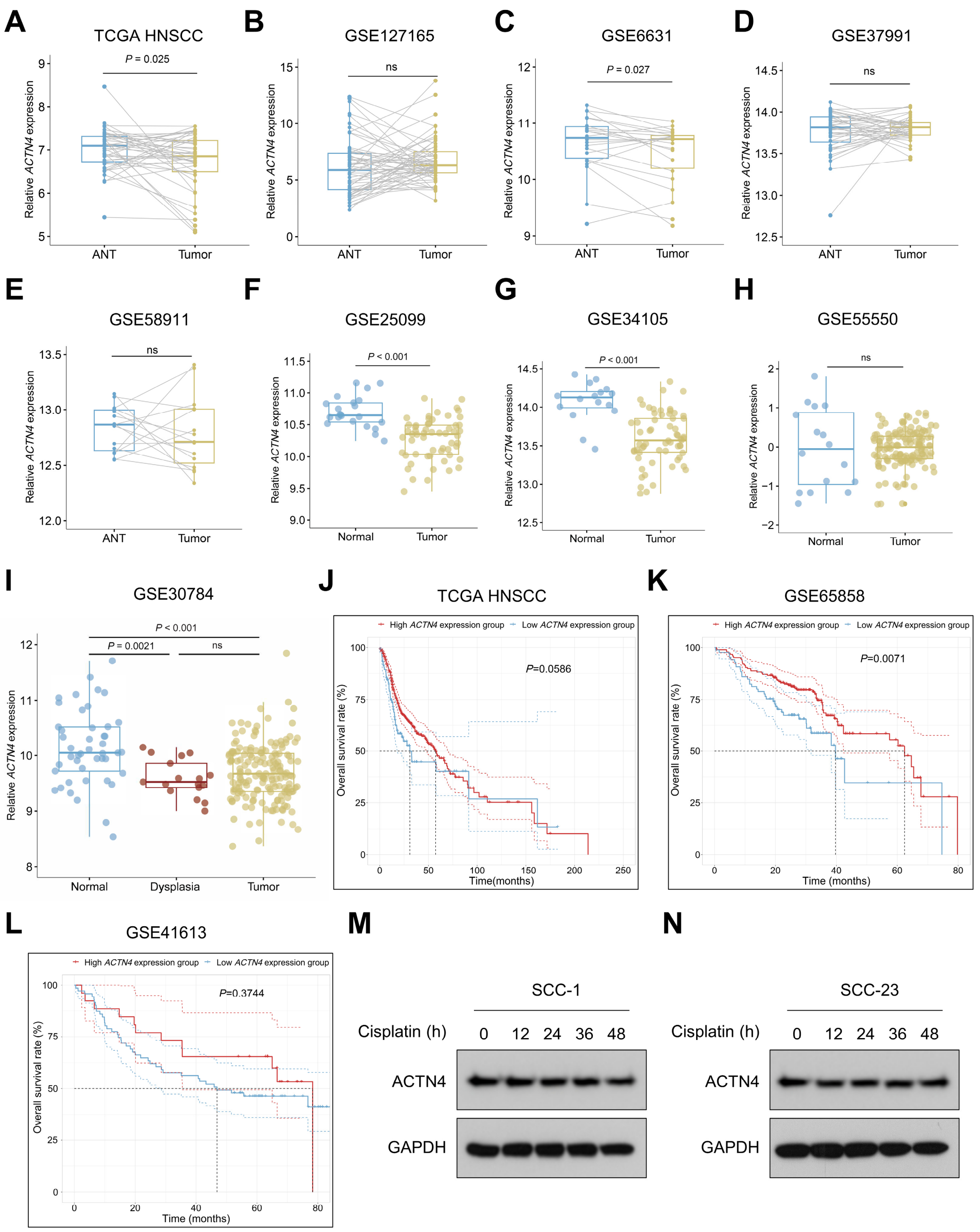

Supplementary Fig. S1 Characterization of *ACTN4* expression profile and its clinical significance in HNSCC. (A-E) Quantification of *ACTN4* expression in the tumor specimens and corresponding ANTs from multiple independent HNSCC cohorts including TCGA HNSCC, GSE127165, GSE6631, GSE37991, and GSE58911. (F-H) Comparative analysis of *ACTN4* expression between tumor and normal tissues using datasets from GSE25099, GSE34105, and GSE55550. (I) Differential *ACTN4* expression in the tumor tissues, dysplasia tissues, and normal tissues as per the GSE30784 dataset. (J-L) Survival probability of HNSCC patients categorized into high *ACTN4* expression and low *ACTN4* expression groups, as revealed by Kaplan-Meier survival analysis using TCGA HNSCC, GSE65858, and GSE41613 datasets. (M-N) Temporal dynamics of ACTN4 protein expression in HNSCC cells subjected to cisplatin exposure over varying durations.

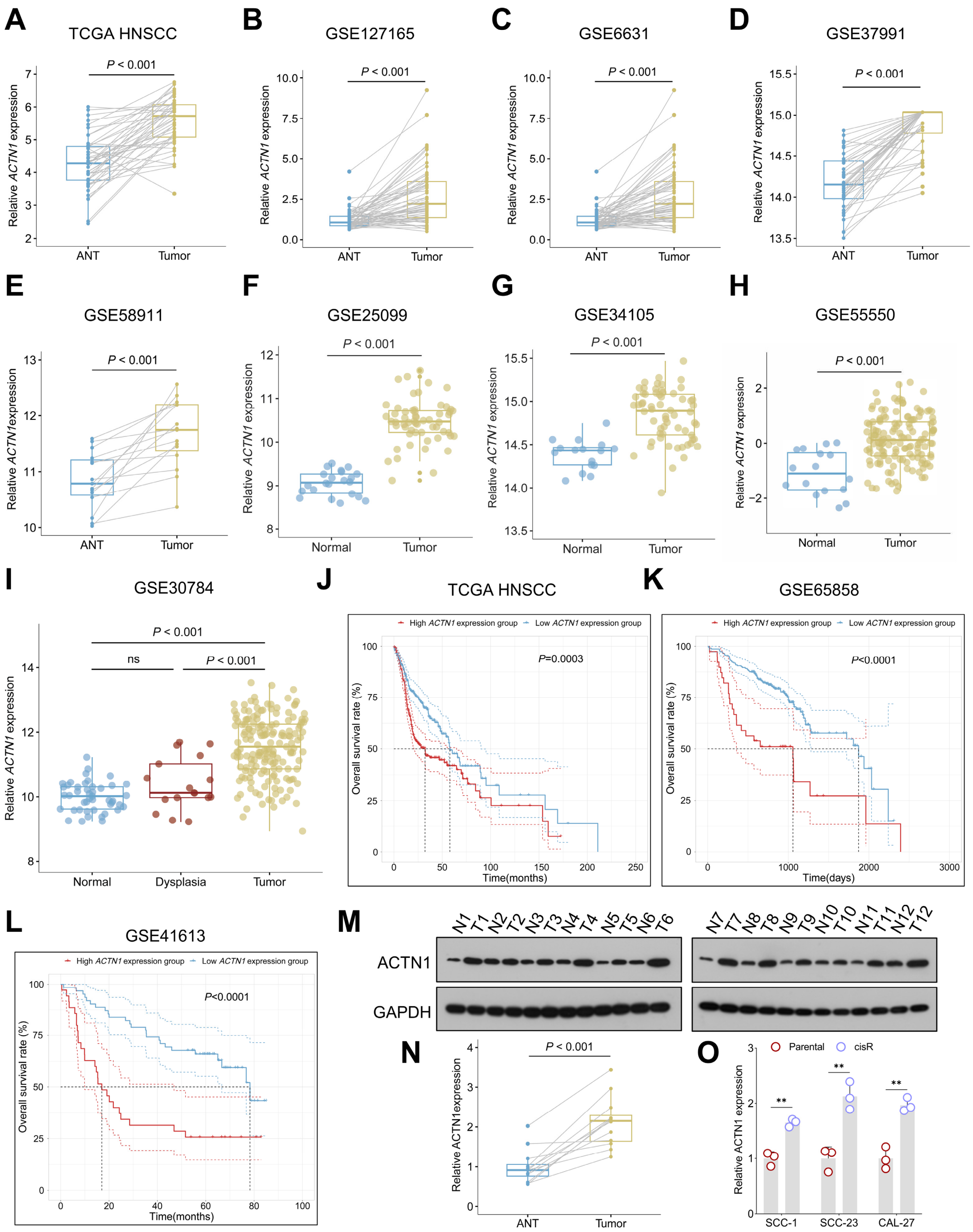

Supplementary Fig. S2 Investigation of *ACTN1* expression patterns and its clinical significance in HNSCC. (A-E) Examination of *ACTN1* expression in the tumor samples and ANTs across multiple independent HNSCC cohorts, including TCGA HNSCC, GSE127165, GSE6631, GSE37991, and GSE58911. (F-H) *ACTN1* expression was analyzed in both tumor and normal tissues in the GSE25099, GSE34105, and GSE55550 datasets. (I) Evaluation of *ACTN1* expression in the tumor tissues, dysplasia tissues, and normal tissues using the GSE30784 dataset. (J-L) Survival analysis based on HNSCC patients with high and low *ACTN1* expression, utilizing datasets from TCGA HNSCC, GSE65858, and GSE41613. (M-N) Expression profile of ACTN1 protein in tumor specimens and ANTs within the in-house HNSCC cohort. (O) Quantitative analysis of the western blot results to compare the expression of ACTN1 between cisplatin-resistant cell lines and their respective controls.

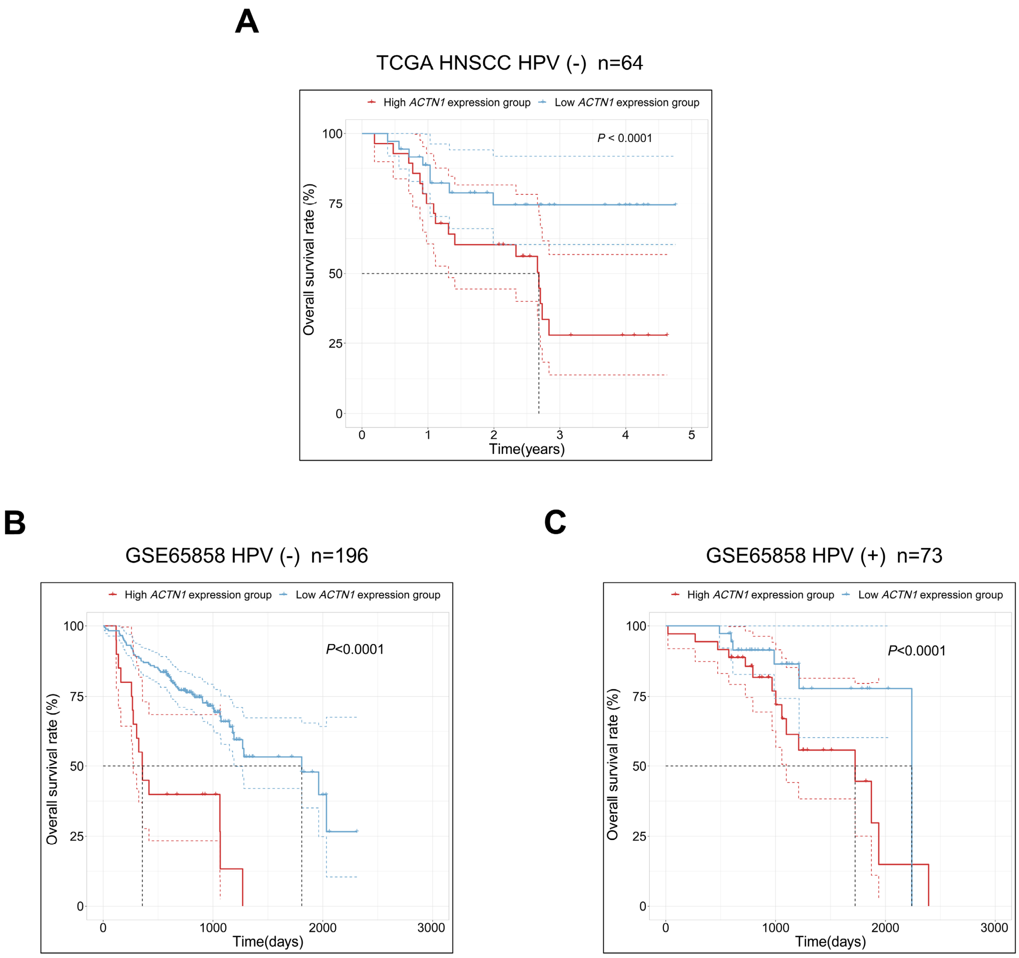

Supplementary Fig. S3 Association of *ACTN1* expression with overall survival in HPV-negative and positive HNSCC. (A) Survival analysis in TCGA HNSCC HPV-negative patients with high and low levels of *ACTN1* expression. (B-C) Survival analysis in GSE65858 HPV-negative and positive patients, categorized by high and low *ACTN1* expression.

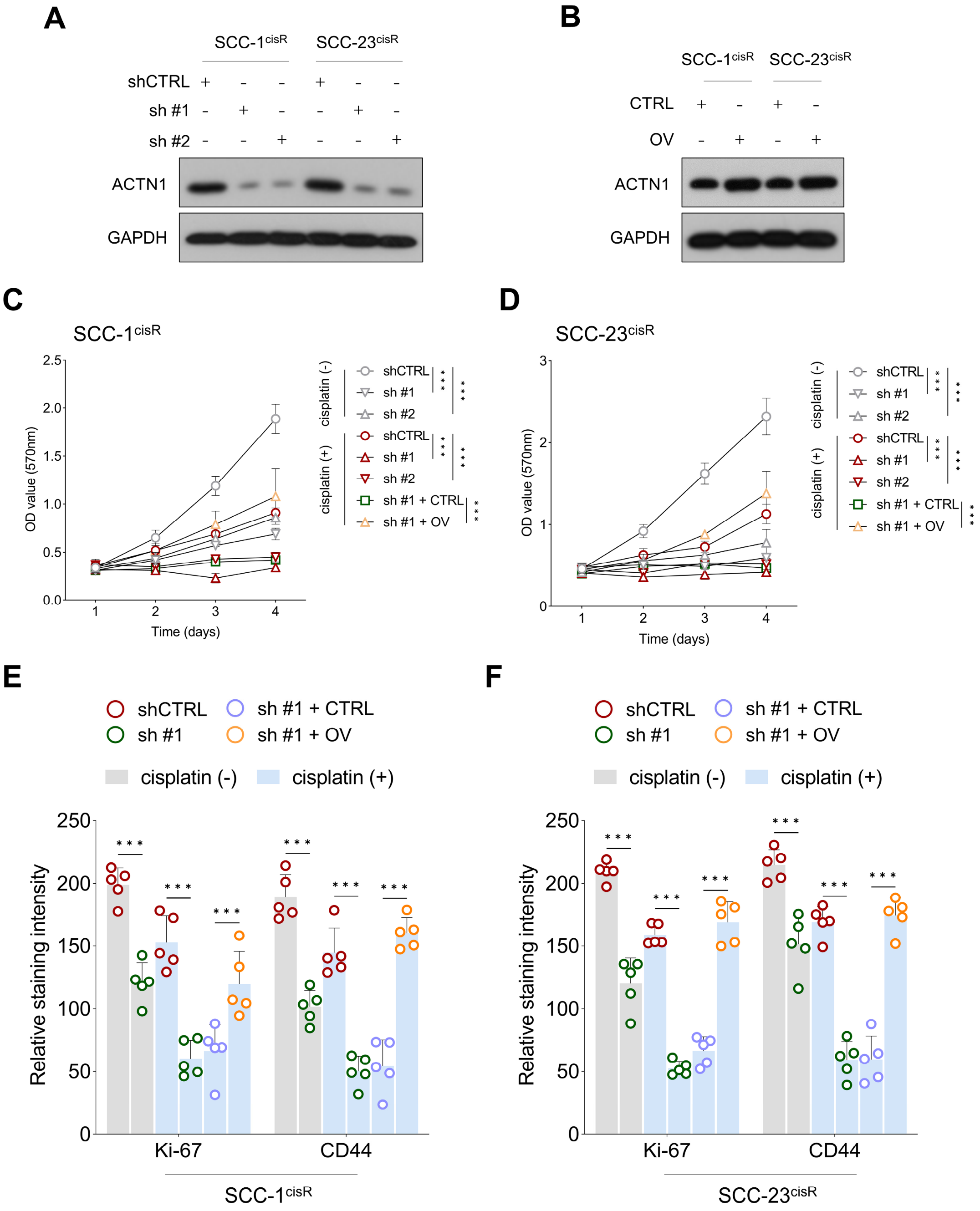

Supplementary Fig. S4 Impact of ACTN1 depletion on malignant phenotypes and cisplatin sensitivity of HNSCC cells *in vitro* and *in vivo*. (A-B) Western blotting analysis of ACTN1 expression in HNSCC cells following transduction with specified lentiviral particles. (C-D) Temporal analysis of optical density values in HNSCC cells subjected to the indicated treatment, as determined by MTT assay. (E-F) Relative staining intensities of Ki-67 and CD44 in xenograft tumor tissues derived from tumor cells exposed to specified treatments. ****P*<0.001.

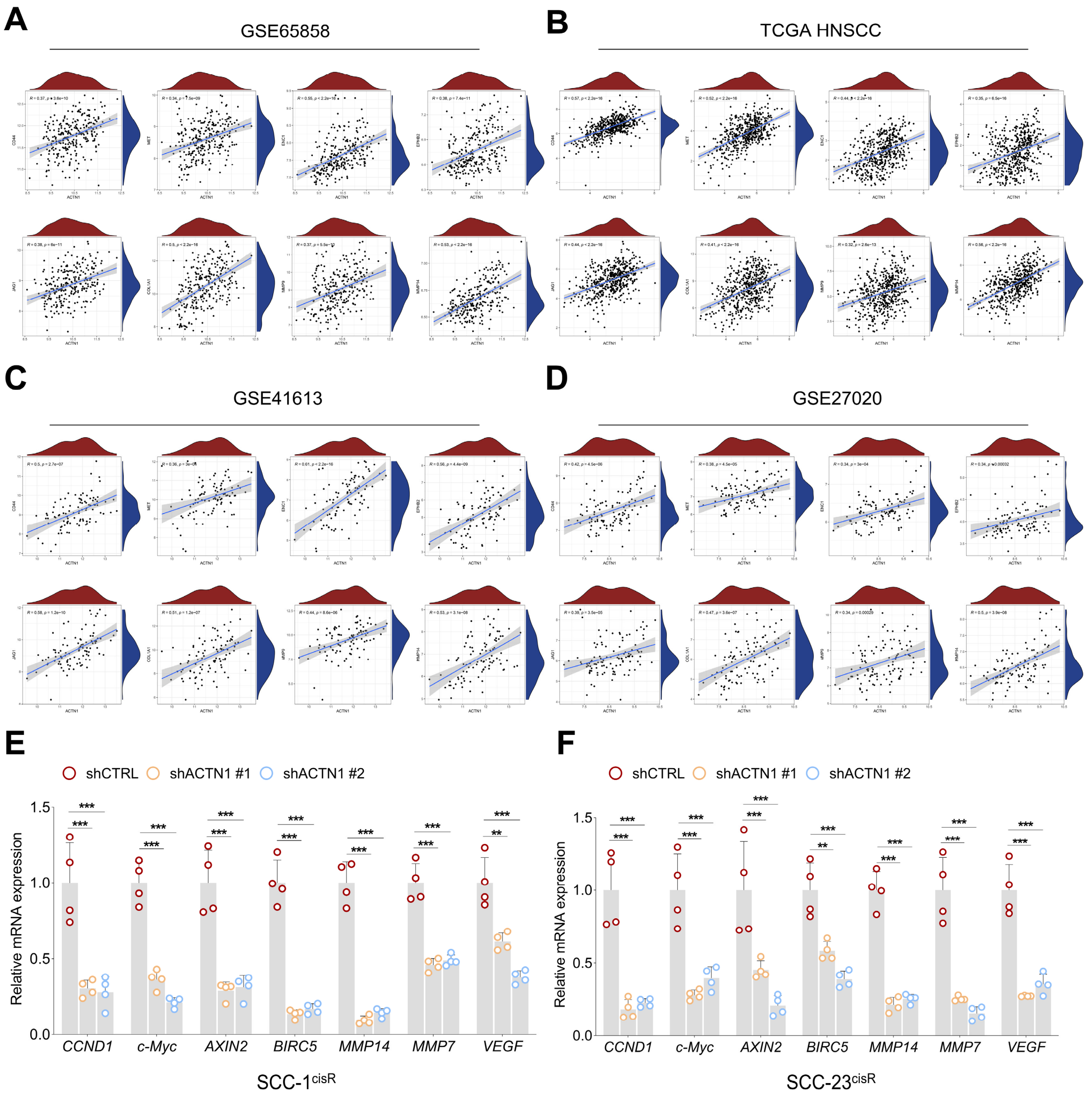

Supplementary Fig. S5 Role of ACTN1 in β-catenin signaling activation in HNSCC cells. (A-D) Correlation analysis between ACTN1 expression and downstream targets of β-catenin signaling across multiple independent HNSCC cohorts, including GSE65858, TCGA HNSCC, GSE41613, and GSE27020. (E-F) qPCR quantification of alterations in β-catenin target gene expression following ACTN1 depletion in HNSCC cells. ***P*<0.01, ****P*<0.001.

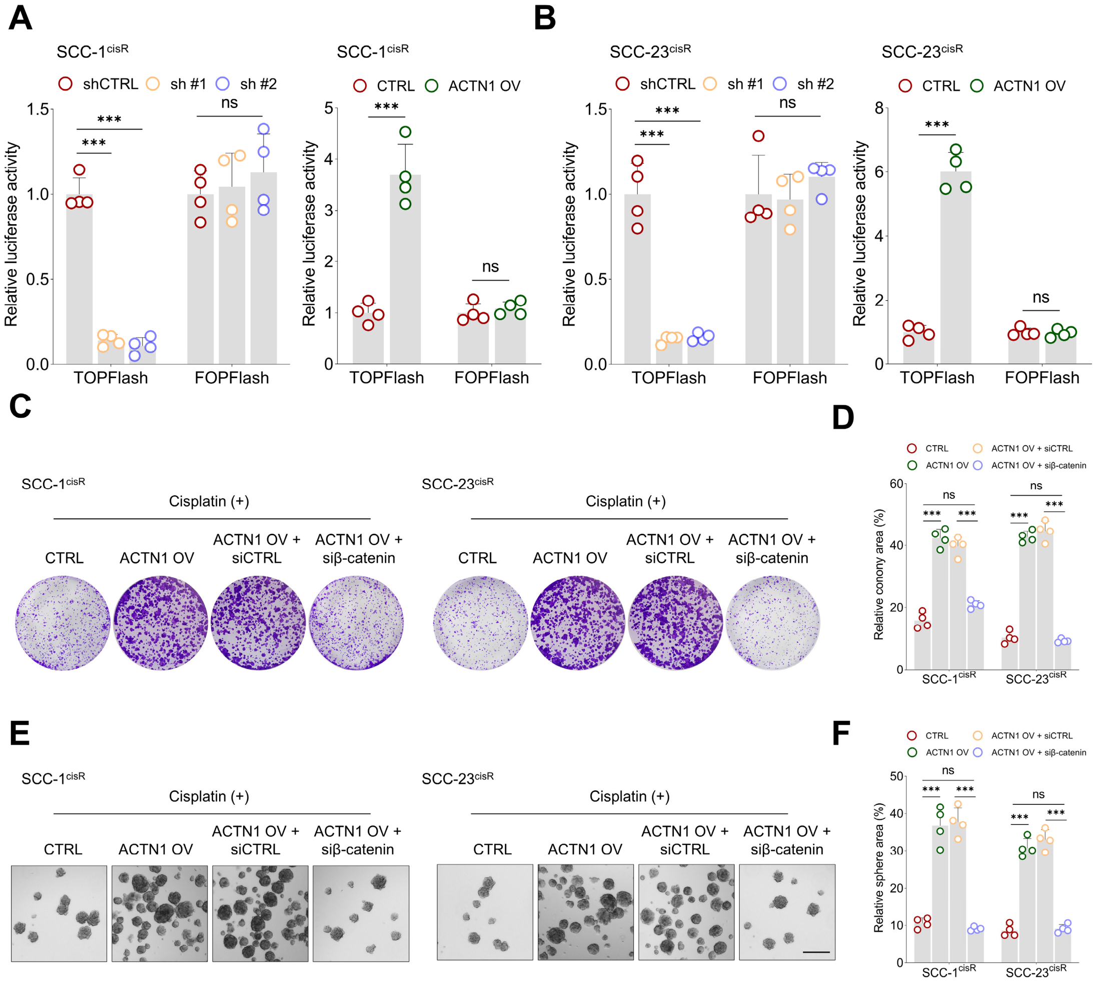

Supplementary Fig. S6 ACTN1 promotes the oncogenic behaviors of HNSCC cells through activating β-catenin signaling. (A) The effect of ACTN1 depletion or overexpression on the relative TOPFlash activity in SCC-1^{cisR} cells. (B) The effect of ACTN1 depletion or overexpression on the relative TOPFlash activity in SCC-23^{cisR} cells. (C-D) Assessment of the colony-forming potential of HNSCC cells following indicated treatments in the presence of cisplatin. (E-F) Evaluation of the sphere formation capabilities of HNSCC cells after specified treatments under cisplatin exposure. ****P*<0.001, ns: not significant.

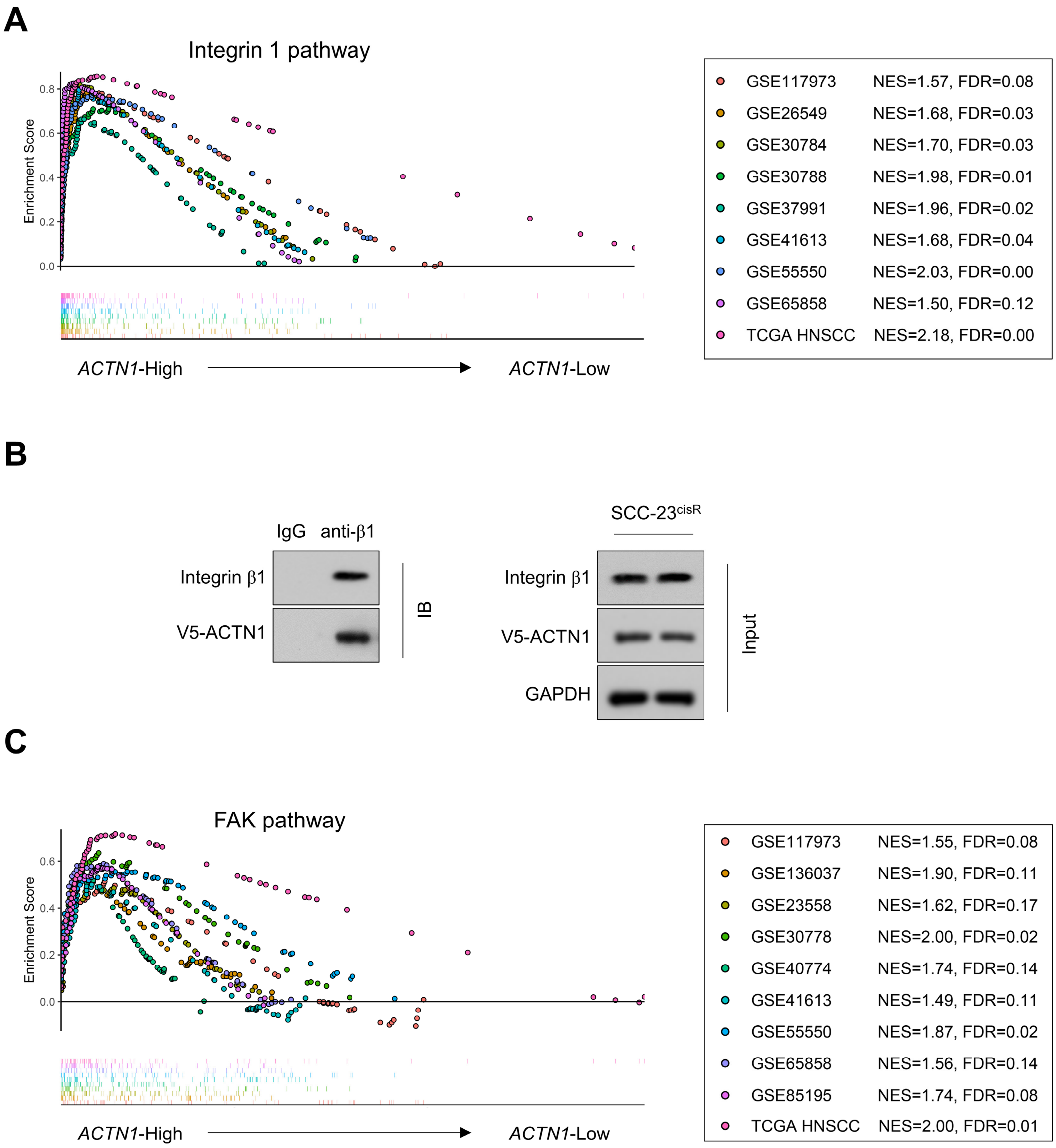

Supplementary Fig. S7 Enrichment of Integrin β1 and FAK pathways in the *ACTN1*-high group across multiple independent HNSCC cohorts. (A) GSEA analysis highlighted the enrichment of the Integrin β1 pathway in the *ACTN1*-high group as analyzed across several HNSCC cohorts. (B) The interaction between integrin β1 and ACTN1 in SCC-23^{cisR} cells, as revealed through Co-IP assay. (C) GSEA analysis underscored the enrichment of the FAK pathway in the *ACTN1*-high group across multiple HNSCC cohorts.

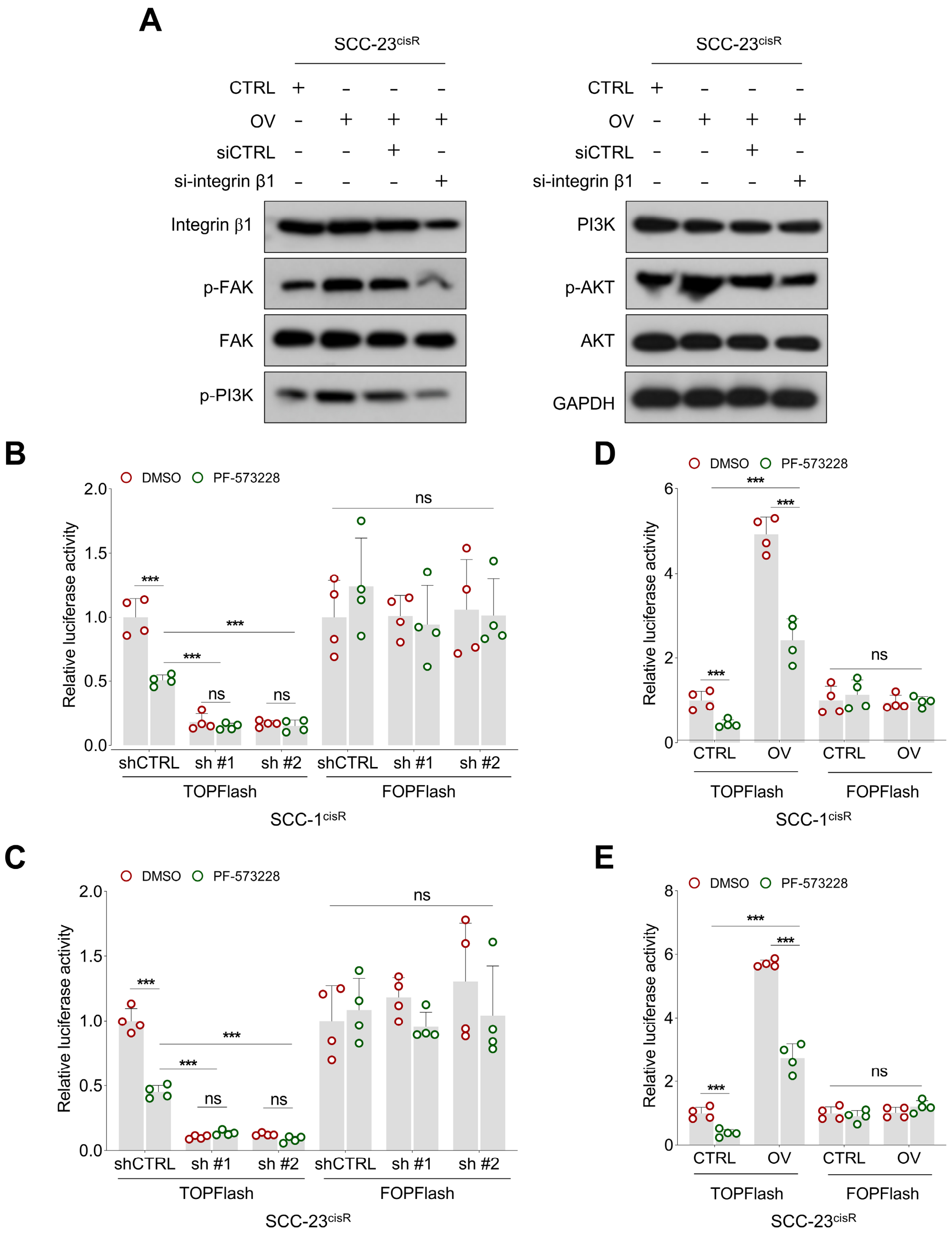

Supplementary Fig. S8 Partial mitigation of ACTN1 overexpression-enhanced β-catenin signaling through FAK inhibition. (A) Western blotting analysis of the effect of integrin β1 depletion on the expression of p-FAK, FAK, p-PI3K, PI3K, p-AKT, and AKT in ACTN1-overexpressing SCC-23^{cisR} cells. (B-C) Relative TOPFlash luciferase activities were presented for both control cells and ACTN1-depleted cells, with or without PF-573228 treatment. (D-E) Relative TOPFlash luciferase activities were shown for control cells and ACTN1-overexpressing cells, also under conditions with or without PF-573228 treatment. ***P<0.001, ns: not significant.

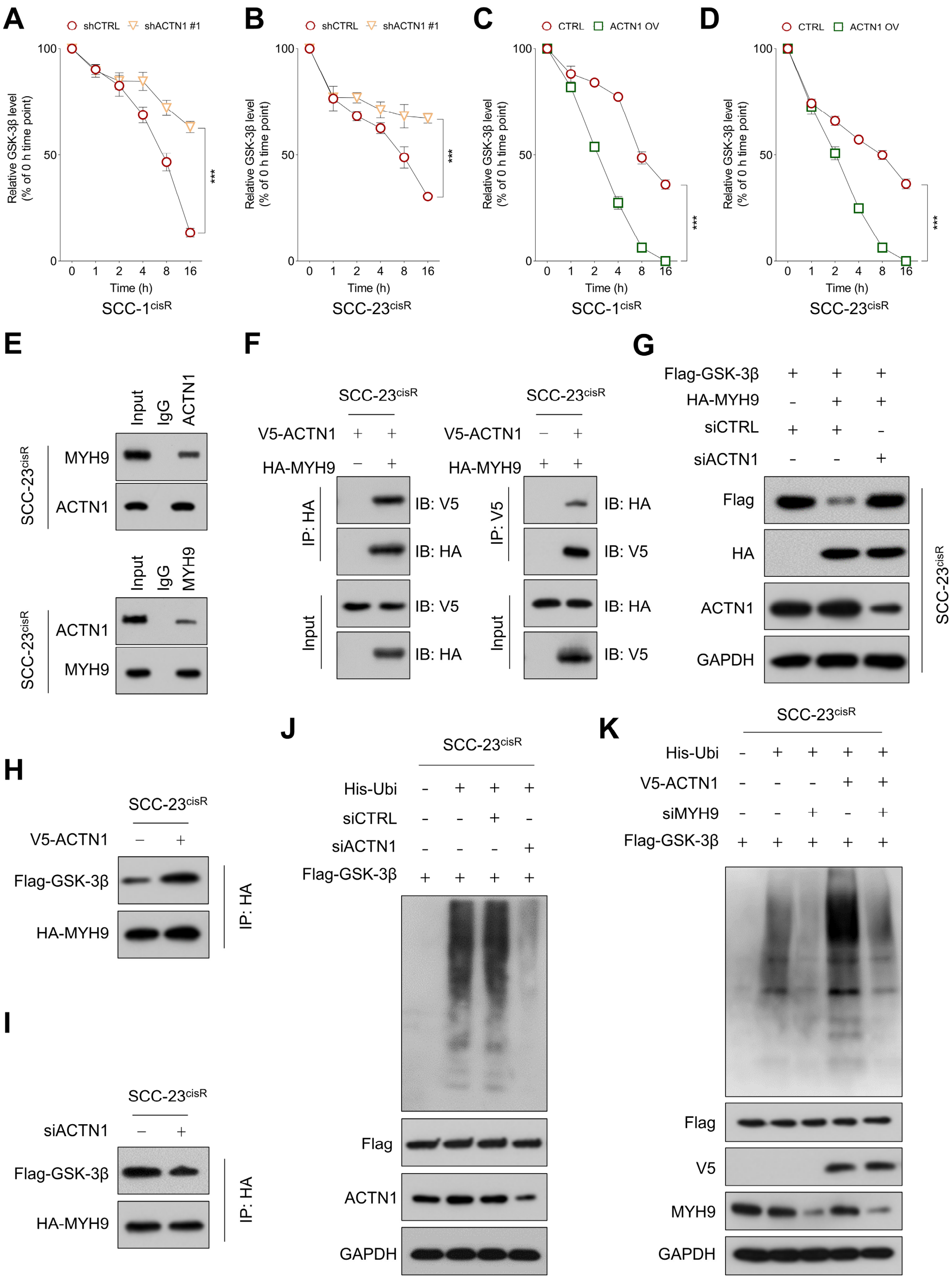

Supplementary Fig. S9 Promotion of GSK-3β degradation by ACTN1 via enhanced interaction with MYH9. (A-B) Assessment of ACTN1 depletion's impact on the degradation rate of GSK-3β. (C-D) Examination of the effects of ACTN1 overexpression on the degradation rate of GSK-3β. (E-F) Exploration of potential interactions between MYH9 and ACTN1, as demonstrated through endogenous and exogenous Co-IP assays. (G) Expression of GSK-3β in SCC-23^{cisR} cells following indicated treatments. (H-I) Effects of ACTN1 overexpression or depletion on the interactions between MYH9 and GSK-3β. (J) Examination of ACTN1 depletion's influence on the ubiquitination level of GSK-3β in SCC-23^{cisR} cells. (K) Influence of ACTN1 overexpression and/or MYH9 depletion on the ubiquitination level of GSK-3β in SCC-23^{cisR} cells. ***P<0.001.

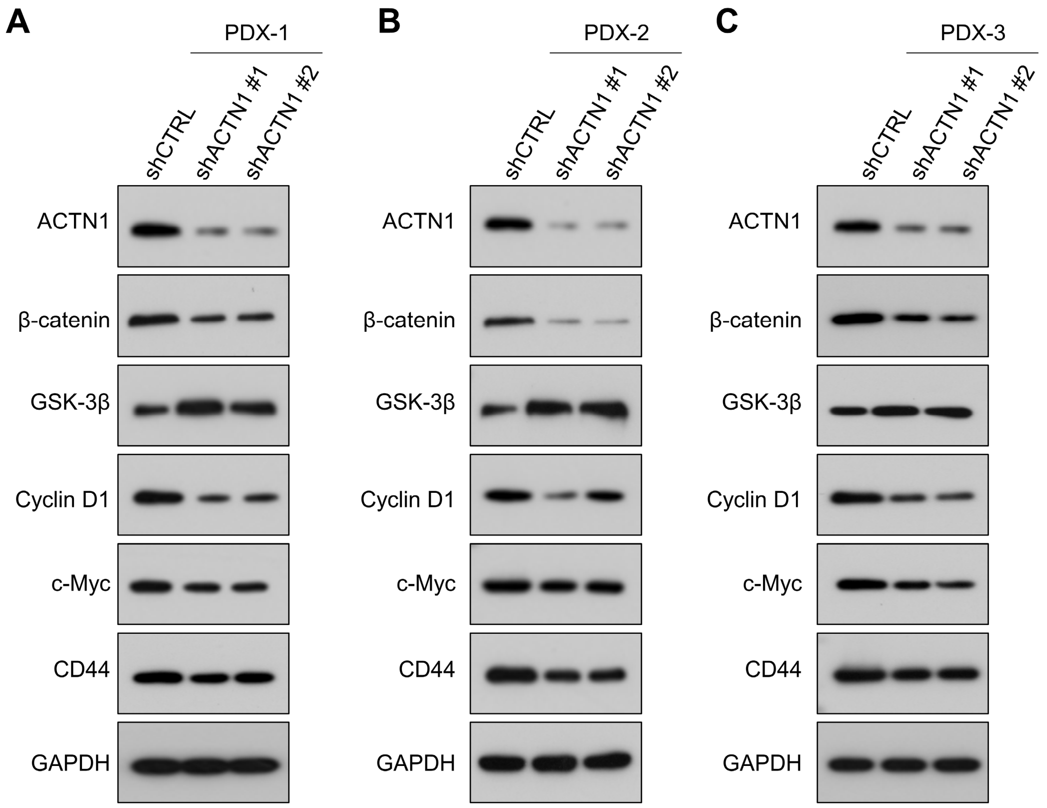

Supplementary Fig. S10 Suppression of β-catenin-mediated signaling in tumor cells from PDX tumors by ACTN1 depletion. (A-C) Assessment of ACTN1 depletion's impact on the expression of GSK-3β, β-catenin, and the downstream targets of β-catenin (cyclin D1, c-Myc, and CD44) in tumor cells isolated from distinct PDX tumors.

Supplementary Table S1. The clinicopathological information of the HNSCC patients.

Clinicopathological parameters	Number of patients (n, %)
**Age**	
≥60	43 (44.79%)
<60	53 (55.21%)
**Gender**	
Male	64 (66.67%)
Female	32 (33.33%)
**Site**	
Oral cavity	59 (61.46%)
Non-oral cavity	37 (38.54%)
**Smoking status**	
Yes	50 (52.08%)
No	46 (47.92%)
**Differentiation**	
G1	48 (50.00%)
G2-G3	48 (50.00%)

Supplementary Table S2. Sequences of primers and oligos used in this study.

Gene	Sequence (5′-3′)
ACTN1	F: CCAAGATTGTCCAGACCTACCAC
	R: CCTCTCATTGTGCTGCTGTCG
CCND1	F: CCCTCGGTGTCCTACTTCAA
	R: AGGAAGCGGTCCAGGTAGTT
MYC	F: AGAGTTTCATCTGCGACCCG
	R: AAGCCGCTCCACATACAGTC
AXIN2	F: CAAACTTTCGCCAACCGTGGTTG
	R: GGTGCAAAGACATAGCCAGAACC
BIRC5	F: TGCCTGGCAGCCCTTTC
	R: CCTCCAAGAAGGGCCAGTTC
MMP14	F: TGCCTACCGACAAGATTGATG
	R: ATCCCTTCCCAGACTTTGATG
MMP7	F: TCGGAGGAGATGCTCACTTCGA
	R: GGATCAGAGGAATGTCCCATACC
VEGF	F: TCACCAAGGCCAGCACATAG
	R: GACAGCAGCGGGCACCAAC
GAPDH	F: TGCACCACCAACTGCTTAGC
	R: GGCATGGACTGTGGTCATGAG
siACTN1	CCACUCACAAAUCUGAAUATT
shACTN1 #1	GCCGAAGACATCGTTGGAACT
shACTN1 #2	GCTGCGACAGAAGGACTATGA

Supplementary Table S3. The correlation of *ACTN1* mRNA expression and immune function in HPV (-) and HPV (+) HNSCC patients.

Comparison Group	HPV (+) Spearman ρ	HPV (+) q-Val.	HPV (+) Signif.	HPV (-) Spearman ρ	HPV (-) q-Val.	HPV (-) Signif.
Aneuploidy Score vs. ACTN1	1.532E-01	8.127E-01	NO	-8.872E-03	3.035E-01	NO
B Cells Memory vs. ACTN1	-1.076E-01	8.174E-01	NO	-1.909E-01	9.318E-04	YES
B Cells Naive vs. ACTN1	-2.434E-01	1.296E-01	NO	-5.420E-02	1.432E-01	NO
BCR Evenness vs. ACTN1	-3.012E-01	9.996E-01	NO	-7.216E-02	7.591E-01	NO
BCR Richness vs. ACTN1	-1.564E-01	2.905E-01	NO	-1.333E-01	2.199E-02	YES
BCR Shannon vs. ACTN1	-1.931E-01	2.244E-01	NO	-1.469E-01	1.307E-02	YES
CTA Score vs. ACTN1	-3.863E-02	7.896E-01	NO	-2.317E-01	3.485E-06	YES
Dendritic Cells vs. ACTN1	7.213E-02	5.141E-01	NO	-1.261E-01	1.583E-02	YES
Dendritic Cells Activated vs. ACTN1	6.426E-02	6.253E-01	NO	-1.064E-01	3.987E-02	YES
Dendritic Cells Resting vs. ACTN1	1.520E-02	9.440E-01	NO	-6.214E-02	2.153E-01	NO
Eosinophils vs. ACTN1	NA	NA	NO	2.381E-02	6.525E-01	NO
Eosinophils 1 vs. ACTN1	NA	NA	NO	2.381E-02	6.525E-01	NO
Fraction Altered vs. ACTN1	6.667E-02	7.990E-01	NO	-1.121E-01	1.130E-02	YES
Homologous Recombination Defects vs. ACTN1	5.694E-02	9.844E-01	NO	-1.646E-01	4.830E-04	YES
IFN gamma Response vs. ACTN1	1.270E-01	6.296E-01	NO	2.270E-01	2.331E-06	YES
Indel Neoantigens vs. ACTN1	1.770E-02	7.537E-01	NO	-1.935E-01	5.366E-03	YES

Intratumor Heterogeneity vs. ACTN1	-3.684E-03	8.308E-01	NO	5.996E-02	1.811E-01	NO
Leukocyte Fraction vs. ACTN1	2.381E-03	7.934E-01	NO	9.714E-02	2.585E-02	YES
Lymphocyte Infiltration Signature Score vs. ACTN1	-2.484E-01	7.911E-02	NO	-8.947E-02	4.312E-02	YES
Lymphocytes vs. ACTN1	-4.105E-01	9.257E-03	YES	-2.842E-01	2.153E-08	YES
Macrophage Regulation vs. ACTN1	-7.448E-02	4.696E-01	NO	1.072E-01	1.271E-02	YES
Macrophages vs. ACTN1	4.127E-01	2.566E-02	YES	2.912E-01	3.268E-08	YES
Macrophages M0 vs. ACTN1	2.247E-01	5.693E-01	NO	2.526E-01	1.215E-06	YES
Macrophages M1 vs. ACTN1	1.713E-01	7.048E-01	NO	1.595E-01	1.772E-03	YES
Macrophages M2 vs. ACTN1	2.410E-01	1.522E-01	NO	-3.790E-02	3.608E-01	NO
Mast Cells vs. ACTN1	-5.124E-02	8.362E-01	NO	8.132E-02	1.928E-01	NO
Mast Cells Activated vs. ACTN1	1.448E-01	6.129E-01	NO	1.477E-01	3.871E-03	YES
Mast Cells Resting vs. ACTN1	-6.746E-02	7.945E-01	NO	-1.290E-01	1.028E-02	YES
Monocytes vs. ACTN1	-9.416E-02	9.504E-01	NO	-1.241E-01	8.030E-02	NO
Neutrophils vs. ACTN1	1.286E-02	9.495E-01	NO	-5.656E-02	3.095E-01	NO
Neutrophils 1 vs. ACTN1	1.286E-02	9.495E-01	NO	-5.656E-02	3.095E-01	NO
NK Cells Activated vs. ACTN1	-3.456E-01	5.086E-02	NO	-6.279E-02	1.468E-01	NO
NK Cells Resting vs. ACTN1	1.768E-01	4.470E-01	NO	1.628E-01	2.336E-03	YES
Nonsilent Mutation Rate vs. ACTN1	-5.221E-02	6.671E-01	NO	-3.238E-01	3.621E-10	YES
Number of Segments vs. ACTN1	1.485E-01	4.803E-01	NO	-1.754E-01	2.329E-04	YES
Plasma Cells vs. ACTN1	-1.201E-01	9.270E-01	NO	-2.631E-01	3.261E-07	YES

Proliferation vs. ACTN1	-9.908E-02	4.420E-01	NO	8.432E-02	4.782E-02	YES
Silent Mutation Rate vs. ACTN1	-7.901E-02	5.378E-01	NO	-2.622E-01	8.302E-07	YES
SNV Neoantigens vs. ACTN1	-1.316E-01	6.178E-01	NO	-3.321E-01	3.672E-10	YES
Stromal Fraction vs. ACTN1	2.567E-02	8.698E-01	NO	2.236E-01	8.117E-06	YES
T Cells CD4 Memory Activated vs. ACTN1	1.296E-02	7.031E-01	NO	-2.964E-02	3.487E-01	NO
T Cells CD4 Memory Resting vs. ACTN1	1.380E-01	1.845E-01	NO	3.164E-01	5.035E-11	YES
T Cells CD4 Naive vs. ACTN1	-8.748E-02	2.488E-01	NO	1.159E-03	4.831E-01	NO
T Cells CD8 vs. ACTN1	-2.044E-01	1.820E-01	NO	-2.134E-01	1.747E-05	YES
T Cells Follicular Helper vs. ACTN1	-1.717E-01	3.409E-01	NO	-3.838E-01	1.772E-14	YES
T Cells gamma delta vs. ACTN1	-1.084E-01	9.996E-01	NO	-8.298E-02	2.242E-01	NO
T Cells Regulatory Tregs vs. ACTN1	-2.126E-01	1.703E-01	NO	-2.068E-01	6.880E-05	YES
TCR Evenness vs. ACTN1	-5.667E-03	8.749E-01	NO	3.888E-02	4.836E-01	NO
TCR Richness vs. ACTN1	-1.438E-01	2.641E-01	NO	5.542E-02	9.171E-02	NO
TCR Shannon vs. ACTN1	-1.277E-01	2.916E-01	NO	3.639E-02	1.725E-01	NO
TGF beta Response vs. ACTN1	5.013E-01	7.503E-05	YES	4.974E-01	5.664E-28	YES
Th1 Cells vs. ACTN1	1.864E-01	3.661E-01	NO	2.464E-01	5.995E-07	YES
Th17 Cells vs. ACTN1	3.858E-01	7.365E-03	YES	1.975E-01	1.307E-04	YES
Th2 Cells vs. ACTN1	2.369E-01	7.341E-02	NO	2.734E-01	2.827E-08	YES
Wound Healing vs. ACTN1	-1.250E-01	1.876E-01	NO	7.331E-02	4.111E-02	YES

Supplementary Table S4. The proteins interacting with ACTN1 identified by mass spectrometry

UniProt No.	Gene symbol	Protein name	Protein_Qscore	Unique_Peptide_Num
P35579	MYH9	Myosin-9	318.13	76
P15924	DSP	Desmoplakin	210.94	58
Q15149	PLEC	Plectin	154.71	40
Q08211	DHX9	ATP-dependent RNA helicase A	104.57	25
Q07157	TJP1	Tight junction protein ZO-1	98.35	24
P51114	FXR1	RNA-binding protein FXR1	96.25	17
Q14258	TRIM25	E3 ubiquitin/ISG15 ligase TRIM25	92.42	18
P14923	JUP	Junction plakoglobin	89.83	18
P08670	VIM	Vimentin	85.61	17
P11142	HSPA8	Heat shock cognate 71 kDa protein	82.78	14

Supplementary Table S5. The tumor-initiating capacity of cancer cells isolated from PDXs with indicated modifications.

Cells injected	Tumor incidence/number of injection			
	shCTRL	cisplatin	shACTN1 #1	shACTN1 #1 + cisplatin
10^5	5/5	5/5	4/5	1/5
10^4	5/5	5/5	3/5	0/5
CSC frequency	1/12542-1	1/12542-1	1/98790-1/13089	1/3519195-1/70566
